# Exploring the role of RCEO in macrophage-mediated modulation of pulmonary fibrosis

**DOI:** 10.1186/s13020-026-01398-w

**Published:** 2026-05-29

**Authors:** Zhiguo Mao, Xiangke Lin, Xingyi Yang, Ying Liu, Chengfu Han, Shuo Tian, Yagang Song, Hui Zhao, Mingsan Miao

**Affiliations:** 1 Department of Pharmacology, Henan University of Chinese Medicine, No. 156 Jinshui East Road, Zhengdong New District, Zhengzhou, 450046 Henan China; 2 Collaborative Innovation Center of Research and Development on the Whole Industry Chain of Yu-Yao in Henan Province, Zhengzhou, 450046 Henan China; 3 Academy of Chinese Medicine Science, Henan University of Chinese Medicine, Zhengzhou, 450046 Henan China

**Keywords:** RCEO, PF, Macrophages, Pi3k/Akt/β-catenin pathway, Tlr4/Myd88/Nf-κb pathway

## Abstract

**Background:**

Pulmonary fibrosis (PF) is an interstitial lung disease with macrophage polarization playing a critical role. Previous research has demonstrated that essential oil from *Radix Curcumae* (RCEO) significantly ameliorates pulmonary sarcoidosis, an effect attributed to the modulation of macrophage polarization. However, the therapeutic potential of RCEO in PF and its underlying mechanisms remain to be elucidated.

**Purpose:**

To investigate whether RCEO ameliorates pulmonary function in PF mice by suppressing the polarization of M1 and M2 macrophages via suppressing the Pi3k/Akt/β-catenin and Tlr4/Myd88/Nf-κb signaling pathways.

**Methods:**

Bioinformatics and network pharmacology approaches were employed to predict the potential therapeutic targets of RCEO for PF. The main components of RCEO and the primary constituents of RCEO deposited in lung tissue following inhalation were analyzed using GC–MS. PF models were established both in vivo and in vitro using bleomycin. Changes in pulmonary function were assessed by whole-body plethysmography. HE staining and Masson staining were used to observe histopathological alterations and fibrosis progression in lung tissues. ELISA was performed to measure changes in fibrosis-related mediators in blood. Flow cytometry was utilized to investigate macrophage polarization and apoptosis levels in lung tissues. Immunofluorescence analysis was applied to quantify the expression and distribution of Tlr4 and Pi3k, as well as their fluorescence co-localization with macrophages, in lung tissues. PCR and WB were conducted to evaluate changes in mRNA expression levels and protein abundance associated with the Tlr4/Myd88/Nf-κb and Pi3k/Akt/β-catenin signaling pathways in lung tissues.

**Results:**

Bioinformatics and Network Pharmacology analyses predicted that the therapeutic effect of RCEO on PF is associated with the modulation of macrophage differentiation via the Tlr4/Myd88/Nf-κb pathway and the Pi3k/Akt/β-catenin signaling pathway. In a PF mouse model, RCEO significantly alleviated pathological alterations and collagen deposition in lung tissues and improved pulmonary function. Results from both in vivo and in vitro flow cytometry demonstrated that RCEO concurrently suppressed the differentiation of macrophages into both M1 and M2 phenotypes, promoted apoptosis in 3T3 cells while inhibiting apoptosis in MLE-12 cells, and reduced the levels of profibrotic mediators. Further PCR and Western blot analyses indicated that RCEO modulates macrophage polarization and extracellular matrix expression by inhibiting the Tlr4/Myd88/Nf-κb and Pi3k/Akt/β-catenin signaling pathways. Immunofluorescence assays revealed that RCEO effectively downregulated the expression of Tlr4 and Pi3k in macrophages, thereby exerting a protective effect against pulmonary fibrosis.

**Conclusion:**

RCEO ameliorates PF by suppressing both M1 and M2 polarization of macrophages via inhibition of the Pi3k/Akt/β-catenin and Tlr4/Myd88/Nf-κb signaling pathways. This leads to reduced release of pro-inflammatory and pro-fibrotic cytokines, decreased extracellular matrix (ECM) deposition, and overall attenuation of fibrotic progression.

**Graphical abstract:**

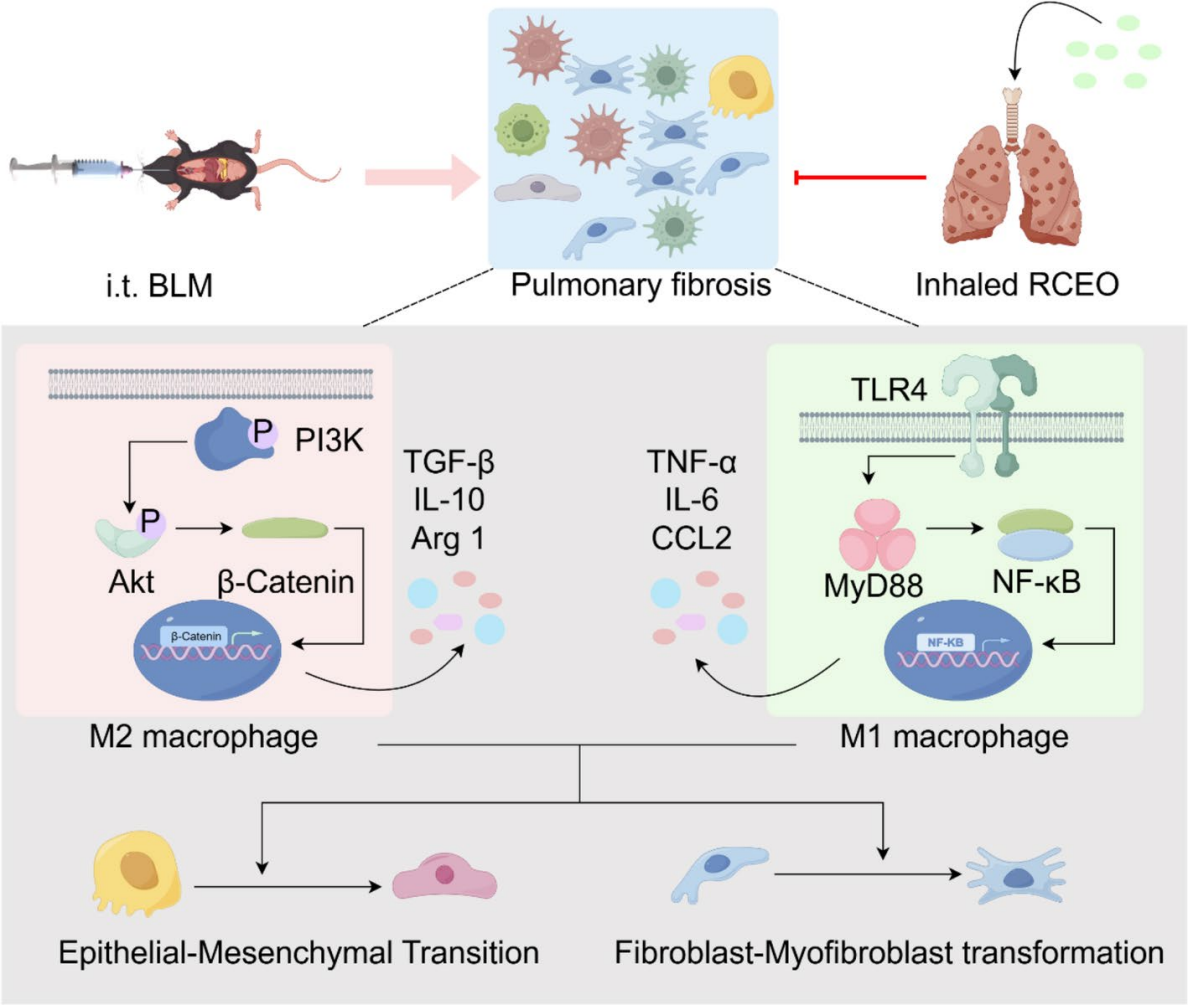

**Supplementary Information:**

The online version contains supplementary material available at 10.1186/s13020-026-01398-w.

## Introduction

Pulmonary fibrosis (PF) is a chronic, irreversible interstitial lung disease predominantly characterized by excessive deposition of the extracellular matrix (ECM) and aberrant tissue repair processes [1, 2]. Risk factors for PF include aging, environmental pollutants, and genetic susceptibility. The clinical manifestations typically involve significant impairment of lung function and dyspnea. The median survival time for patients with PF is less than five years [3–6]. Currently, first-line pharmacological treatments approved by the U.S. Food and Drug Administration (FDA), such as nintedanib and pirfenidone, have been shown to effectively slow disease progression and enhance patient survival and quality of life; however, they do not provide a cure [7, 8]. Therefore, elucidating the pathophysiological mechanisms of PF and developing novel therapies are urgently needed.

The progression of PF is characterized by a complex interplay of biological processes, notably immune cell infiltration—primarily by macrophages—as well as epithelial-to-mesenchymal transition (EMT) and fibroblast-to-myofibroblast transition (FMT). The latter two processes are significant contributors to ECM deposition [9–11]. As the primary defense mechanism against foreign antigens, macrophages are integral to the pathogenesis of PF [12]. Research has demonstrated that the Toll-like receptor 4 (Tlr4)/myeloid differentiation factor 88 (Myd88)/nuclear factor kappa B (Nf-κb) signaling pathway is a well-established cascade that induces the polarization of M1 macrophages, which are classically activated. These M1 macrophages secrete chemokines such as chemokine (C–C motif) ligand 2 (Ccl2), which recruit circulating monocytes and thereby exacerbate the progression of PF [13, 14]. Conversely, the Phosphoinositide 3-kinase (Pi3k)/protein kinase B (Akt)/β-catenin pathway facilitates the polarization of M2 macrophages, which are alternatively activated and secrete factors such as transforming growth factor-beta (Tgf-β), promoting EMT and FMT, and further aggravating PF [15, 16]. Some studies propose that both M1 and M2 macrophages are present during PF development, rather than exclusively M2 macrophages [17, 18]. Nevertheless, the specific role of M1 macrophages in PF remains a subject of debate. Consequently, further research is necessary to elucidate whether M1 and M2 macrophages collaboratively contribute to PF, and this hypothesis requires experimental validation in murine models.

Essential oils derived from Traditional Chinese Medicine (TCM) offer significant benefits in the management of chronic lung diseases. Inhalational administration facilitates direct drug delivery to lung tissues, thereby minimizing systemic exposure and associated adverse effects. Essential oil from *Radix Curcumae* (RCEO), obtained from the dried tuberous roots of *Curcuma wenyujin*, *Curcuma phaeocaulis*, *Curcuma kwangsiensis*, and *Curcuma longa*, exhibits notable antioxidant, anti-inflammatory, immunomodulatory, and antimicrobial properties. It has demonstrated efficacy in treating lung diseases, including lung cancer and pulmonary nodules [19, 20]. Our preliminary investigations have shown that RCEO inhalation significantly reduces pulmonary inflammation and enhances lung function in a murine model of pulmonary nodules [21]. Nonetheless, the therapeutic effects and underlying mechanisms of RCEO in PF remain unexplored. Our recent findings suggest that RCEO substantially improves lung function in PF mice. Bioinformatics, network pharmacology, PCR, and WB analyses indicate its involvement in the “Pi3k/Akt signaling pathway” and the “TLR signaling pathway.” We hypothesize that the therapeutic effects of RCEO may be linked to macrophage polarization, regulated by the Pi3k/Akt/β-catenin and Tlr4/Myd88/Nf-κb pathways. This study aims to elucidate the therapeutic potential and underlying mechanisms of RCEO in PF.

## Methods

### Gene expression omnibus (GEO) analysis

RNA-seq data from normal and PF samples were sourced from publicly accessible datasets (GSE53845, GSE24206, GSE35145) available in the GEO database (https://www.ncbi.nlm.nih.gov/geo/). The batch effects arising from different batches within the same platform and dataset were removed using the “removeBatchEffect” function from the limma package in R. During the data preprocessing stage, boxplots were employed to assess the normalization status of the data, and principal component analysis (PCA) plots were used to evaluate the removal of batch effects. For datasets that were not pre-normalized, a log2 transformation was consistently applied. Differential mRNA expression analysis was performed using the Limma package (version 3.40.2) within the R software environment. To account for false-positive results, adjusted P-values were calculated. The criteria for identifying differentially expressed mRNAs were set at an adjusted P-value less than 0.05 and an absolute log2(fold change) greater than 1. To further investigate the pathogenic roles of the target genes, Gene Ontology (GO) functional annotation and Kyoto Encyclopedia of Genes and Genomes (KEGG) pathway enrichment analyses were performed using the ClusterProfiler package in R. Statistical analyses were executed using R software (version 4.0.3), with a P-value of less than 0.05 deemed statistically significant [22].

### Preparation of RCEO

Approximately 500 g of *Radix Curcumae* was purchased from Tongrentang (Beijing, China; 230,401) and ground into a coarse powder. The volatile oil was extracted using a supercritical fluid extractor (Spe-ed SFE-2, Agilent Technologies, USA), resulting in a dark purple viscous liquid, which was subsequently sealed and stored at − 20 °C.

### Gas chromatography-mass spectrometry (GC–MS) analysis

The composition of the RCEO was analyzed in accordance with a previously established method [21]. GC–MS was conducted using an Agilent system (USA) equipped with a DB-5 quartz capillary column (30 m × 250 μm × 0.1 μm). The analytical conditions were as follows: the carrier gas was helium (99.999%) at a flow rate of 1 mL/min; the split injector temperature was set at 270 °C with a split ratio of 10:1; and the injection volume was 1 μL. The temperature program was configured as follows: an initial temperature of 50 °C was maintained for 2 min, then increased to 90 °C at a rate of 20 °C/min, followed by an increase to 150 °C at 2.5 °C/min (held for 5 min), and finally raised to 250 °C at 10 °C/min (held for 7 min). The electron impact (EI) ion source was operated at 70 eV, with an ion source temperature of 200 °C and a transfer line temperature of 260 °C. The scan range was set between 35 and 550 amu. The components of the RCEO were identified by comparing the mass spectra with those in the NIST mass spectral library.

### Network pharmacology and molecular docking analysis

Potential targets of RCEO were identified utilizing the SwissTargetPrediction (http://www.swisstargetprediction.ch/) and Super-PRED (https://prediction.charite.de/subpages/target_prediction.php) platforms. The target identification strategy for the Swiss Target Prediction database was set to Probability > 0, while for the Super PRED database, it was set to Probability > 70%. Targets associated with PF were sourced from the GeneCards (https://www.genecards.org) and OMIM (https://omim.org/) databases. The disease search keyword was “pulmonary fibrosis”. The Wei Sheng Xin platform (https://www.bioinformatics.com.cn/) was utilized to screen therapeutic targets of RCEO relevant to PF. A protein–protein interaction (PPI) network was developed using the STRING database (https://cn.string-db.org). Gene Ontology (GO) enrichment and KEGG pathway analyses of the core targets were conducted via the DAVID database (https://davidbioinformatics.nih.gov/). Visualization tasks were performed using the Wei Sheng Xin platform (https://www.bioinformatics.com.cn/) [23, 24].

Structural data for drug molecules were acquired from PubChem (https://pubchem.ncbi.nlm.nih.gov/), and protein 3D coordinates were retrieved from the Protein Data Bank (PDB, http://www.rcsb.org/). The 3D coordinates for PI3K are 7RNU, and for TLR4, they are 2Z62. Molecular docking studies between drug molecules and pathway targets were visualized using CB-Dock 2 (https://cadd.labshare.cn/cb-dock2/php/index.php) [22, 25].

### Cellular thermal shift assay (CETSA)

The CETSA was performed as previously described [26]. In summary, RAW 264.7 murine macrophages were exposed to germacrone (100 μM, Yuanye, China) or to dimethyl sulfoxide (DMSO, GC203006, Servicebio, China) as a control, within a CO_2_ incubator maintained at 37 °C for a duration of 12 h, after which the cells were harvested. The harvested cells were lysed using RIPA buffer supplemented with protease and phosphatase inhibitors. The resulting lysate was partitioned into eight aliquots, each subjected to heating at progressively increasing temperatures (37, 42, 47, 52, 57, 62, 67, and 72 °C) for 3 min. This was followed by three freeze–thaw cycles utilizing liquid nitrogen. Subsequently, centrifugation was performed at 4 °C for 15 min, and the supernatants were collected. The levels of Pi3k and Tlr4 were then quantified using equivalent amounts of protein.

### Animals

Male C57BL/6 mice, aged six to eight weeks and weighing between 18.0 and 22.0 g (n = 150), were procured from Shandong Pengyue Laboratory Animal Breeding Co., Ltd., holding License No. SCXK (Lu) 20,220,006. These specific pathogen-free (SPF) mice were maintained under controlled conditions, including a temperature of 22 °C, humidity at 55%, and a 12-h light/dark cycle, with unrestricted access to food and water. All experimental protocols received approval from the Experimental Animal Ethics Committee of Henan University of Chinese Medicine (IACUC-202405027) and adhered strictly to the ethical standards outlined in the NIH Guide for the Care and Use of Laboratory Animals.

### PF model and drug treatment

The PF mouse model was established as previously described [27]. Bleomycin (BLM, JS249810, Yuanye, China) powder was dissolved in phosphate-buffered saline (PBS) to create a BLM suspension. On the initial day of modeling, a 50 μL aliquot of the BLM suspension (3 mg/kg) was administered via intratracheal injection into the lung tissue [28, 29]. The procedure for intratracheal injection was as follows: mice in the model group were anesthetized with sodium pentobarbital (50 mg/kg), positioned supine on the operating table, and had their neck hair removed and disinfected with iodophor. A 1 cm longitudinal incision was made along the midline of the neck, followed by blunt dissection with forceps to expose the trachea. After the intratracheal injection of the 50 μL BLM suspension, the incision was promptly sutured. Subsequently, the mice were rotated laterally and suspended in a head-up position for 1 min to ensure uniform distribution of the BLM suspension within the lung tissue.

In Protocol 1, mice were randomly allocated into five distinct groups: the control group, the model group, the pirfenidone (PFD, S23HS195349, Yuanye, China) group, the high-dose RCEO (RCEO-10) group, and the low-dose RCEO (RCEO-5) group. Seven days after BLM injection, mice in the RCEO groups received intranasal inhalation of RCEO at doses of 0.1 mL/kg (high-dose) or 0.05 mL/kg (low-dose) once daily. The positive control group was treated with a 300 mg/kg PFD solution via oral gavage [30] once daily, whereas the model and control groups were given intranasal olive oil once daily for a duration of 14 consecutive days. In Protocol 2, mice were randomly assigned to eight groups: the control group, the model group, the RCEO group, the RS 09 (Tlr4 agonist, 120,588, MCE, China) group, the 740-Y-P (Pi3k agonist, 514,611, MCE, China) group, the RCEO + RS 09 (RR) group, the RCEO + 740-Y-P (R7) group, and the RCEO + RS 09 + 740-Y-P (RR7) group. Seven days post-BLM injection, RCEO was administered via intranasal inhalation at a dose of 0.1 mL/kg once daily. RS 09 (10 mg/kg) [31] and 740-Y-P (100 mg/kg) [32] were delivered through intraperitoneal injection commencing on the second day post-modeling. The model and control groups received intranasal olive oil once daily for 14 consecutive days.

### Cell culture, co-culture system, and treatment

RAW 264.7 cells (mouse monocyte-macrophage leukemia cells, CL-0190, Procell, China), 3T3-L1 cells (mouse embryonic fibroblasts, CL-0006, Procell, China), and MLE-12 cells (mouse alveolar epithelial cells, FH1103, Fuheng Biology, China) were cultured in Dulbecco's Modified Eagle Medium (DMEM, PM150210A, Procell, China) supplemented with 10% fetal bovine serum (FBS, S711-050S, LONSERA, China) at 37 °C in a 5% CO₂ atmosphere. Differentiation of RAW 264.7 cells was achieved using 50 μg/mL BLM. MLE-12 and 3T3-L1 cells were maintained in basal DMEM medium. The supernatant from RAW 264.7 cells was combined in a 1:1 ratio with the basal medium to create a conditioned medium, which was then applied to MLE-12 or 3T3-L1 cells for a duration of 24 h. Subsequently, RAW 264.7, MLE-12, and 3T3-L1 cells were harvested for further experimental analyses. In Experiment 1, RAW 264.7 cells were subjected to various treatments for 24 h at 37 °C in a 5% CO₂ environment prior to the collection of cells and supernatant. The treatment groups included a control group, a BLM group, and BLM groups supplemented with RCEO at concentrations of 20 μg/mL, 40 μg/mL, and 80 μg/mL. In Experiment 2, RAW 264.7 cells were cultured for 24 h at 37 °C in a 5% CO₂ atmosphere, subjected to the following treatment groups prior to the collection of cells and supernatants: Control group, BLM group, BLM + RCEO (80 μg/mL, BR) group, BLM + RCEO + RS 09 (5 μg/mL, BRR) group, BLM + RCEO + 740-Y-P (15 μM, BR7) group, and BLM + RCEO + RS 09 + 740-Y-P (BRR7) group.

### Pulmonary function assessment

After a 14-day treatment period, pulmonary function was assessed using a non-invasive pulmonary function testing system (EMKA, China) to measure tidal volume (TV), respiratory frequency (f), and peak expiratory flow (PEF).

### Hematoxylin–eosin (HE) and masson staining

Lung tissues were fixed in 10% neutral formalin, embedded in paraffin, and sectioned at 4 μm thickness. Following deparaffinization, the sections were rehydrated and subjected to histological analysis. Pathological alterations were evaluated using hematoxylin and eosin (HE) and Masson staining, with imaging conducted via a slide scanner (KFBIO, China).

### Enzyme-linked immunosorbent assay (ELISA)

Additionally, after 14 days of treatment, blood samples were collected from the abdominal aorta following a 12-h fasting period. Serum was isolated by centrifugation and stored at − 20 °C. The concentrations of Tgf-β (JL13959), Tumor Necrosis Factor-alpha (Tnf-α, JL10484), Interleukin-1 beta (Il-1β, JL18442), and Interleukin-6 (Il-6, JL20268) were quantified in accordance with the manufacturer's instructions (JONLNBIO, China).

### Hydroxyproline determination

A hydroxyproline (HYP) assay kit (BC0255, Solarbio, China) was employed to quantify the hydroxyproline content in lung tissues. The right upper lung lobe was accurately weighed and subjected to hydrolysis in 5% sodium hydroxide for 20 min. Following pH adjustment to a range of 6.0–6.8, the hydrolysate underwent centrifugation at 3,500 rpm for 10 min, and the supernatant was subsequently measured at 560 nm using a microplate reader.

### Immunohistochemical staining

Post tissue fixation, lung sections were embedded, sectioned, deparaffinized, and subjected to antigen retrieval. The sections were incubated overnight at 4 °C with primary antibodies targeting E-cadherin (1:3000, GB12083-100, Servicebio, China) or α-smooth muscle actin (α-sma, 1:1000, GB111364-100, Servicebio, China), followed by washing and incubation with biotinylated secondary antibodies for 20 min. The slides were developed using a DAB working solution, counterstained with hematoxylin, and examined under a bright-field upright microscope.

### RNA extraction and quantitative real-time polymerase chain reaction (qRT-PCR)

Total RNA was isolated from the samples utilizing the Animal Tissue Total RNA Extraction Kit (G3640, Servicebio, China). Subsequently, 50 ng of the isolated RNA was reverse-transcribed into complementary DNA (cDNA) according to the protocol specified in the SweScript All-in-One RT SuperMix for qPCR Kit (G3337, Servicebio, China). Quantitative real-time PCR (qRT-PCR) analysis was conducted using the SYBR qPCR Master Mix (G3328, Servicebio, China) on the QuantStudio 6 FLEX system (ABI, USA). The data were analyzed employing the relative quantification method (2^−ΔΔCT^). The primer sequences utilized are detailed in Table 1.

**Table 1:** Primer sequences used for qRT-PCR

Genes		Primer sequence	Product Size (bp)
*Pik3r*	S	5'- TTGACAGTAGGAGGAGGTTGGA-3'	212
	A	5'- CAGGGAGTATTGATCTTCGGTATT-3'	
*Akt*	S	5'- GCTGCTCAAGAAGGACCCTAC-3'	116
	A	5'- GCTTCTTCTCATACACATCCTGC −3'	
*β-catenin*	S	5'- TGCAGTTGCTTTATTCTCCCAT-3'	161
	A	5'- TGTTGCCACGCCTTCATTC-3'	
*Tlr4*	S	5'-TGAGGACTGGGTGAGAAATGAGC-3'	223
	A	5'-CTGCCATGTTTGAGCAATCTCAT-3'	
*Myd88*	S	5'-GATGCCTTTATCTGCTACTGCC-3'	174
	A	5'-CATGCGGCGACACCTTTT-3'	
*Nf-κb*	S	5'-GAGTCACGAAATCCAACGCAG-3'	90
	A	5'-CGTCATCACTCTTGGCACAATC-3'	
*Il-10*	S	5'-CAACATACTGCTAACCGACTCCTT-3'	81
	A	5'-AACTGGATCATTTCCGATAAGGC-3'	
*Arg1*	S	5'-ATCAACACTCCCCTGACAACCA-3'	255
	A	5'-TTCCATCACCTTGCCAATCC-3'	
*Inos*	S	5'-AGCTCGGGTTGAAGTGGTATG-3'	245
	A	5'-CACAGCCACATTGATCTCCG-3'	
*Tnf-α*	S	5'-ACCCTCACACTCACAAACCA-3'	212
	A	5'-ATAGCAAATCGGCTGACGGT-3'	
*Il6*	S	5'-CCCCAATTTCCAATGCTCTCC-3'	141
	A	5'-CGCACTAGGTTTGCCGAGTA-3'	
*α-sma*	S	5'-GTACCACCATGTACCCAGGC-3'	152
	A	5'-GAAGGTAGACAGCGAAGCCA-3'	
*E-cadherin*	S	5'-TCCAACAGGGACAAAGAAACAA-3'	171
	A	5'-TGACACGGCATGAGAATAGAGG-3'	
*Collagen i*	S	5'-CTCCCAGAACATCACCTATCACT-3'	220
	A	5'-GGAGGTCTTGGTGGTTTTGTATT-3'	
*Fibronectin*	S	5'-AAGGCTGGATGATGGTGGACT-3'	140
	A	5'-TCGGTTGTCCTTCTTGCTCC-3'	
*β-Actin*	S	5'-GTGACGTTGACATCCGTAAAGA-3'	287
	A	5'-GTAACAGTCCGCCTAGAAGCAC-3'	

### Western blotting

Following PBS washing, lung tissues and cells were lysed using RIPA buffer (R0010, Solarbio, China). Subsequent centrifugation at 12,000 rpm for 15 min allowed for the collection of supernatants. The protein lysates were then dissolved in 1 × SDS buffer and subjected to separation via SDS-PAGE. Proteins were transferred onto PVDF membranes (0000354260, Millipore, USA), which were incubated with primary antibodies at 4 °C overnight. This was followed by incubation with secondary antibodies at room temperature for 1 h. Signal detection was performed using an enhanced chemiluminescence kit (SQ201, Epizyme Biotech, China) after three washes. The primary antibodies utilized included: anti-Tlr4 (1:2000, 19,811–1-AP Proteintech, China), anti-Myd88 (1:2000, 29,946–1-AP, Proteintech, China), anti-Nf-κb (1:3000, 10,745–1-AP, Proteintech, China), anti-p-Nf-κb (1:5000, 82,335–1-RR, Proteintech, China), anti-Inos (1:2000, 18,985–1-AP, Proteintech, China), anti-Il-10 (1:2000, 82,191–3-RR, Proteintech, China), anti- arginase-1 (Arg-1, 1:10,000, 16,001–1-AP, Proteintech, China), anti-Tnf-α (1:5000, 80,258–6-RR, Proteintech, China), anti-p-Pi3k (1:1000, T40116, Abmart, China), anti-Pi3k (1:1000, T40115, Abmart, China), anti-p-Akt (1:5000, 66,444–1-Ig, Proteintech, China), anti-Akt (1:6000, 10,176–2-AP, Proteintech, China), anti-β-catenin (1:2000, CY3523, Abways, China), anti-E-cadherin (1:2000, CY5288, Abways, China), anti-α-sma (1:5000, CY1132, Abways, China), anti-Collagen-I (1:1000, TA7001, Abmart, China), anti-Fibronectin (1:2000, CY9537, Abways, China), and anti-β-actin (1:6000, 20,536–1-AP, Proteintech, China). β-actin served as the internal control.

### Immunofluorescence

Immunofluorescence staining was executed as previously described [33]. In summary, slides were incubated with mouse-reactive antibodies, including anti-F4/80 (1:5000, GB113373, Servicebio), anti-CD86 (1:400, Servicebio), anti-CD206 (1:800, Servicebio), anti-Tlr4 (1:2000, GB11519, Servicebio), anti-Pi3k (1:1000, GB113360, Servicebio), and anti-Ccr2 (1:5000, GB11326, Servicebio). Following treatment with fluorescent secondary antibodies and DAPI counterstaining, images were acquired using a fluorescence microscope (Thermo Fisher, USA).

### Flow cytometry

Lung tissues were finely minced and subjected to enzymatic digestion using a solution comprising collagenase type I (1.8 mg/mL, C8140, Solarbio, China), DNase I (1 μL/mL, D8071, Solarbio, China), and complete medium, followed by erythrocyte lysis. The isolated lung tissue cells, along with RAW 264.7 cells, were subsequently stained with the following antibodies: APC-conjugated anti-mouse F4/80 antibody (catalog number 123115, BioLegend, USA) and Brilliant Violet 421™-conjugated anti-mouse CD86 antibody (catalog number 105031, BioLegend, USA). After staining for 30 min, RAW 264.7 cells were fixed and permeabilized using the Cyto-Fast™ Fix/Perm Buffer Set, followed by incubation with PE-conjugated anti-mouse CD206 antibody (catalog number 141705, BioLegend, USA) at 4 °C for 30 min. Post-incubation, the cells underwent two washes with PBS supplemented with fetal bovine serum (FBS) and were then resuspended in PBS for flow cytometric analysis using a Beckman Coulter system (USA).

For the cultured MLE-12 or 3T3-L1 cells, apoptosis was evaluated utilizing the Annexin V-AbFluor™ 488/PI Apoptosis Detection Kit (KTA0002, Abbkine, China), following the manufacturer's instructions. The washed cells were resuspended in PBS for subsequent flow cytometry (Beckman Coulter, USA).

### Statistical analysis

Data analysis was conducted using GraphPad Prism version 8.0.2, with results expressed as mean ± standard deviation (SD). Multiple group comparisons were performed using one-way ANOVA followed by Tukey post-hoc test, while comparisons between two groups were conducted using Student's t-test. A *P*-value of less than 0.05 was considered indicative of statistical significance.

## Results

### PF is associated with macrophage-related pathways

**Fig. 1:**
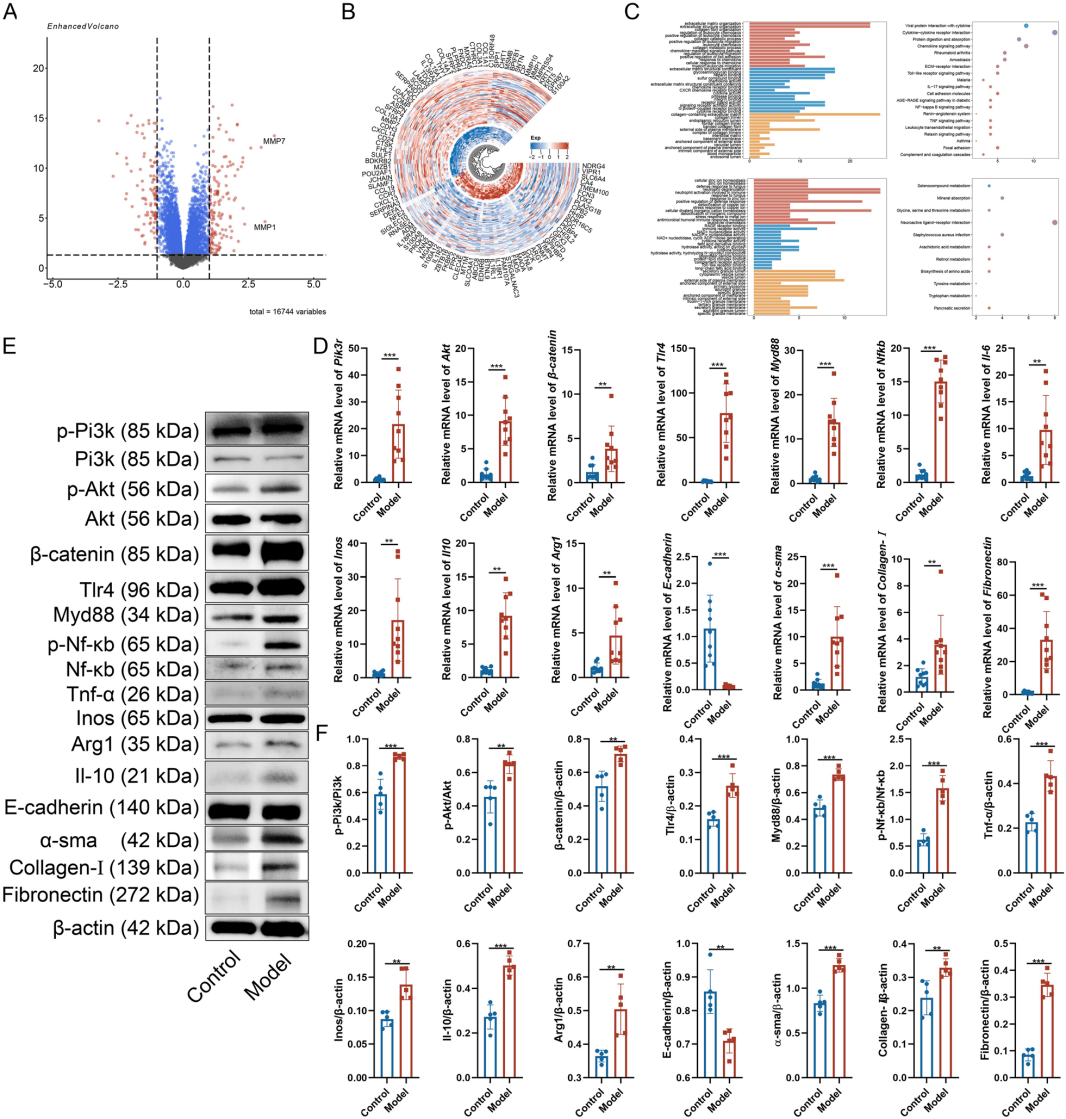
Prediction of potential targets in PF. **A** Volcano plot of differentially expressed genes (DEGs) between normal and PF samples, with upregulated genes displayed in the upper right quadrant and downregulated genes in the upper left quadrant. **B** Heatmap showing highly expressed genes in red and lowly expressed genes in blue. **C** GO enrichment analysis and KEGG pathway enrichment analysis. **D**–**F** mRNA expression levels (n = 9), protein bands, and quantitative analysis (n = 5) of Pi3k, Akt, β-catenin, Tlr4, Myd88, Nf-κb, Il-6, Inos, Il-10, Arg1, E-cadherin, α-sma, Collagen-Ⅰ, and Fibronectin. Data analysis was performed using Student's t-test and data were presented as mean ± SD. *P < 0.05, **P < 0.01

In order to explore the potential pathophysiological mechanisms underlying PF, differential expression analysis and subsequent enrichment analysis was conducted to identify significantly altered gene sets and functional enrichments between control and PF samples, as documented in GEO accession numbers GSE53845, GSE24206, and GSE35145. The analysis identified a differentially expressed gene set comprising 243 genes, of which 146 were upregulated and 97 were downregulated. GO analysis indicated that PF pathophysiology involves processes such as “extracellular matrix organization,” “chemokine activity,” and “regulation of leukocyte chemotaxis.” Furthermore, KEGG pathway analysis suggested that the progression of PF is linked to the “chemokine signaling pathway,” “Toll-like receptor signaling pathway,” “NF-kappa B signaling pathway,” and “TNF signaling pathway” (Fig. 1A–C).

**Fig. 2:**
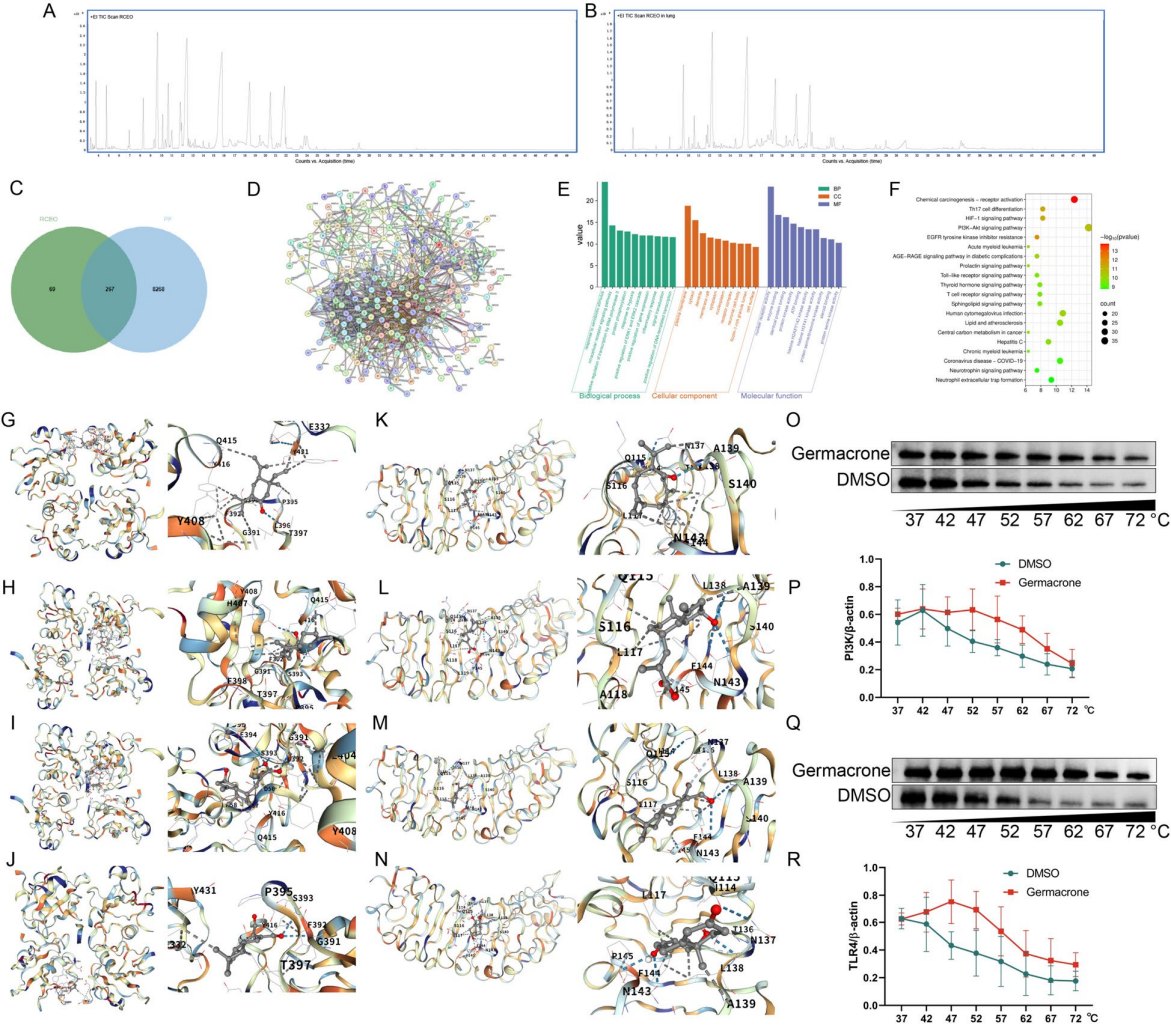
Predicted mechanisms underlying RCEO treatment for PF. **A** Total ion chromatogram of RCEO analyzed by GC–MS. **B** Total ion chromatogram of RCEO in lung tissue analyzed by GC–MS. **C** Common targets of RCEO and PF. **D** PPI network constructed based on shared targets. **E**, **F** Results of GO and KEGG enrichment analyses of shared targets. **G**–**J** Representative molecular docking modes between RCEO components and Pi3k. **K**–**N** Representative molecular docking modes between RCEO components and Tlr4. **O**–**R** Cells were treated with germacrone (100 μM) or DMSO for 12 h and lysed with RIPA buffer. Pi3k and Tlr4 protein levels were determined at 37, 42, 47, 52, 57, 62, 67, and 72 °C, followed by analysis via WB (n = 5). Data analysis was performed using one-way ANOVA and data are presented as mean ± SD; *P < 0.05, **P < 0.01

To elucidate the material basis of RCEO, its chemical composition was analyzed using GC–MS, resulting in the identification of 24 major components, with germacrone, curzerene, beta-elemene, velleral, and beta-elemenone being the most prominent. To identify which RCEO components reach the lungs, volatile compounds were extracted from the lung tissue of RCEO-treated mice and subjected to identical GC–MS analysis, identifying 22 major chemical constituents, with germacrone, curzerene, velleral, beta-elemenone, and beta-elemene as the top five. A comparative analysis revealed 17 overlapping compounds, which are considered the pharmacologically active constituents of RCEO (Fig. 2A, B, Tables 2, 3).

**Table 2:** Components of RCEO

Serial number	Retention time (min)	Component	Molecular formula	CAS	Peak area proportion (%)
1	3.261	(-)-Beta-pinene	C10H16	18,172-67-3	0.4
2	3.776	Eucalyptol	C10H18O	470-82-6	2.29
3	4.785	Camphor	C10H16O	464-49-3	2.17
4	6.985	(-)-Bornyl acetate	C12H20O2	5655-61-8	0.65
5	8.337	Cyclohexene	C15H24	20,307-84-0	2.42
6	9.711	BETA-ELEMENE	C15H24	515-13-9	10.81
7	10.199	BETA-CARYOPHYLLENE	C15H24	87-44-5	1.61
8	10.459	Gamma-muurolene	C15H24	30,021–74-0	0.64
9	10.742	Gamma-elemene	C15H24	3242-08-8	4.27
10	11.08	Humulene	C15H24	6753-98-6	0.89
11	11.905	Cis-beta-copaene	C15H24	18,252-44-3	3.34
12	12.011	BETA-SELINENE	C15H24	17,066–67-0	1.26
13	12.543	Curzerene	C15H20O	17,910-09-7	16.35
14	12.681	Delta-guaiene	C15H24	3691-11-0	0.51
15	13.313	Gamma-gurjunene	C15H24	22,567-17-5	0.63
16	15.917	Germacrone	C15H22O	6902-91-6	21.63
17	18.577	beta-elemenone	C15H22O	20,303-60-0	9.19
18	19.547	Chiapin B	C19H26O6	33,649-17-1	1.06
19	20.566	Hydroxyvalerenic acid	C15H22O3	1619-16-5	5.87
20	20.688	Beta-cyclocostunolide	C15H20O2	2221-82-1	0.56
21	21.935	Velleral	C15H20O2	50,656-61-6	9.74
22	22.063	Cyclobutane, tetrakis(1-methylethylidene)-	C16H24	88,919-66-8	0.61
23	23.858	(±)−5c,8c-dihydroxy-4a-methyl-(4ar,4bt,8at)−4,4a,4b,5,6,7,8,8a,9,10-decahydro-3H-phenanthren-2-one	C15H22O3	94,327-62-5	1.34
24	24.08	Alantic anhydride	C15H20O2	80,367-94-8	1.78

**Table 3:** Components of RCEO in mouse lung tissue after RCEO inhalation

Serial number	Retention time (min)	Component	Molecular formula	CAS	Peak area proportion (%)
1	4.757	(+)-Camphor	C10H16O	464-49-3	1.01
2	8.287	delta-eIemene	C15H24	20,307-84-0	0.75
3	9.6	BETA-ELEMENE	C15H24	515-13-9	7.28
4	10.132	BETA-CARYOPHYLLENE	C15H24	87-44-5	0.79
5	10.658	Gamma-elemene	C15H24	3242-08-8	2.38
6	11.817	Gamma-muurolene	C15H24	30,021–74-0	1.3
7	11.95	BETA-SELINENE	C15H24	17,066–67-0	2.02
8	12.393	Curzerene	C15H20O	17,910-09-7	15.87
9	14.626	Caryophyllene oxide	C15H24O	1139-30-6	0.65
10	15.751	Germacrone	C15H22O	6902-91-6	25.68
11	17.125	Atractylon	C15H20O	6989-21-5	0.84
12	17.74	Alpha-eudesmol	C15H26O	473-16-5	2.34
13	18.062	Cyperenone	C15H22O	3466-15-7	2.72
14	18.461	Beta-elemenone	C15H22O	20,303-60-0	10.53
15	19.502	Chiapin B	C19H26O6	33,649-17-1	1.15
16	20.289	Alpha-cyperone	C15H22O	473-08-5	1.56
17	20.466	Hydroxyvalerenic acid	C15H22O3	1619-16-5	7.08
18	20.616	Beta-cyclocostunolide	C15H20O2	2221-82-1	0.58
19	21.791	Velleral	C15H20O2	50,656-61-6	10.85
20	21.979	Cyclobutane, tetrakis(1-methylethylidene)-	C16H24	88,919-66-8	1.24
21	23.797	(±)−5c,8c-dihydroxy-4a-methyl-(4ar,4bt,8at)−4,4a,4b,5,6,7,8,8a,9,10-decahydro-3H-phenanthren-2-one	C15H22O3	94,327-62-5	1.52
22	24.035	Alantic anhydride	C15H20O2	80,367-94-8	1.85

In addition, network pharmacology was utilized to elucidate the potential mechanisms underlying the ameliorative effects of RCEO on PF by identifying prospective targets and associated pathways. As depicted in the Venn diagram, RCEO was associated with 336 potential biological targets, whereas PF was linked to 8,535 potential targets, with an overlap of 267 shared targets. Analyses of protein–protein interaction (PPI) networks, GO enrichment, and KEGG pathways were conducted based on these overlapping targets. The PPI network analysis identified “PIK3CA,” “PIK3R1,” “TLR4,” “NFKB1,” “TNF,” and “IL6” as central nodes within the network. GO enrichment analysis suggested that the therapeutic effects of RCEO are associated with biological processes such as “inflammatory response” and “protein serine kinase activity.” KEGG pathway analysis indicated the involvement of the “Pi3k-Akt signaling pathway” and the “Toll-like receptor signaling pathway” in the ameliorative effects of RCEO on PF. Collectively, these bioinformatics and network pharmacology findings propose that the therapeutic effects of RCEO may be mediated through the regulation of inflammation via the “Pi3k-Akt signaling pathway” and the “Toll-like receptor signaling pathway.” (Fig. 2C–F). To substantiate these findings, PCR and WB analyses were conducted to evaluate alterations in gene and protein expression within the lung tissues of PF mouse models. The results indicated a significant upregulation of genes and proteins associated with the “Pi3k-Akt signaling pathway” and the “Toll-like receptor signaling pathway” in fibrotic lungs. Furthermore, fibroblast-related genes, including α-sma, Collagen-I, and Fibronectin, demonstrated markedly increased mRNA and protein expression levels, whereas the expression of the alveolar epithelial cell marker, E-cadherin, was significantly downregulated. Notably, both pro-inflammatory factors (Il-6, Inos) and anti-inflammatory factors (Arg1, Il-10) were upregulated, suggesting simultaneous pro- and anti-inflammatory responses during pulmonary fibrosis progression (Fig. 1D–F).

To elucidate the regulatory role of RCEO in the “Pi3k-Akt signaling pathway” and the “Toll-like receptor signaling pathway,” molecular docking and CETSA were employed to assess the binding affinity of RCEO's active constituents with the pathway-initiating proteins Pi3k and Tlr4. The results indicated that eight components of RCEO exhibited strong binding affinity with PI3K (Alantic anhydride-PI3K = − 7.2 kcal/mol, beta-Cyclocostunolide-PI3K = − 7.1 kcal/mol, beta-Elemenone-PI3K = − 6.5 kcal/mol, Germacrone-PI3K = − 6.9 kcal/mol, Hydroxyvalerenic Acid-PI3K = − 7.2 kcal/mol, Velleral-PI3K = − 6.5 kcal/mol, Chiapin B-PI3K = − 8.3 kcal/mol, (±)−5c,8c-dihydroxy-4a-methyl-(4ar,4bt,8at)−4,4a,4b,5,6,7,8,8a,9,10-decahydro-3H-phenanthren-2-one-PI3K = − 8.0 kcal/mol), characterized by binding energies of ≤ − 6.0 and the presence of hydrogen bonds, while five components demonstrated high affinity for TLR4 (Germacrone-TLR4 = − 6.4 kcal/mol, (±)−5c,8c-dihydroxy-4a-methyl-(4ar,4bt,8at)−4,4a,4b,5,6,7,8,8a,9,10-decahydro-3H-phenanthren-2-one-TLR4 = − 6.2 kcal/mol, Hydroxyvalerenic Acid-TLR4 = − 6.3 kcal/mol, Velleral-TLR4 = − 6.0 kcal/mol, Chiapin B-TLR4 = − 6.2 kcal/mol). CETSA further corroborated the interaction of germacrone (the most abundant component in RCEO) with these critical proteins, revealing a significant enhancement in the thermal stability of PI3K and TLR4 in samples treated with germacrone compared to those treated with DMSO controls (Fig. 2G–R). These findings imply that these signaling pathways may contribute to the therapeutic effects of RCEO, with germacrone, hydroxyvalerenic acid, and velleral identified as potential key components targeting both pathways.

### Effects of RCEO on pulmonary function in fibrotic mice

**Fig. 3:**
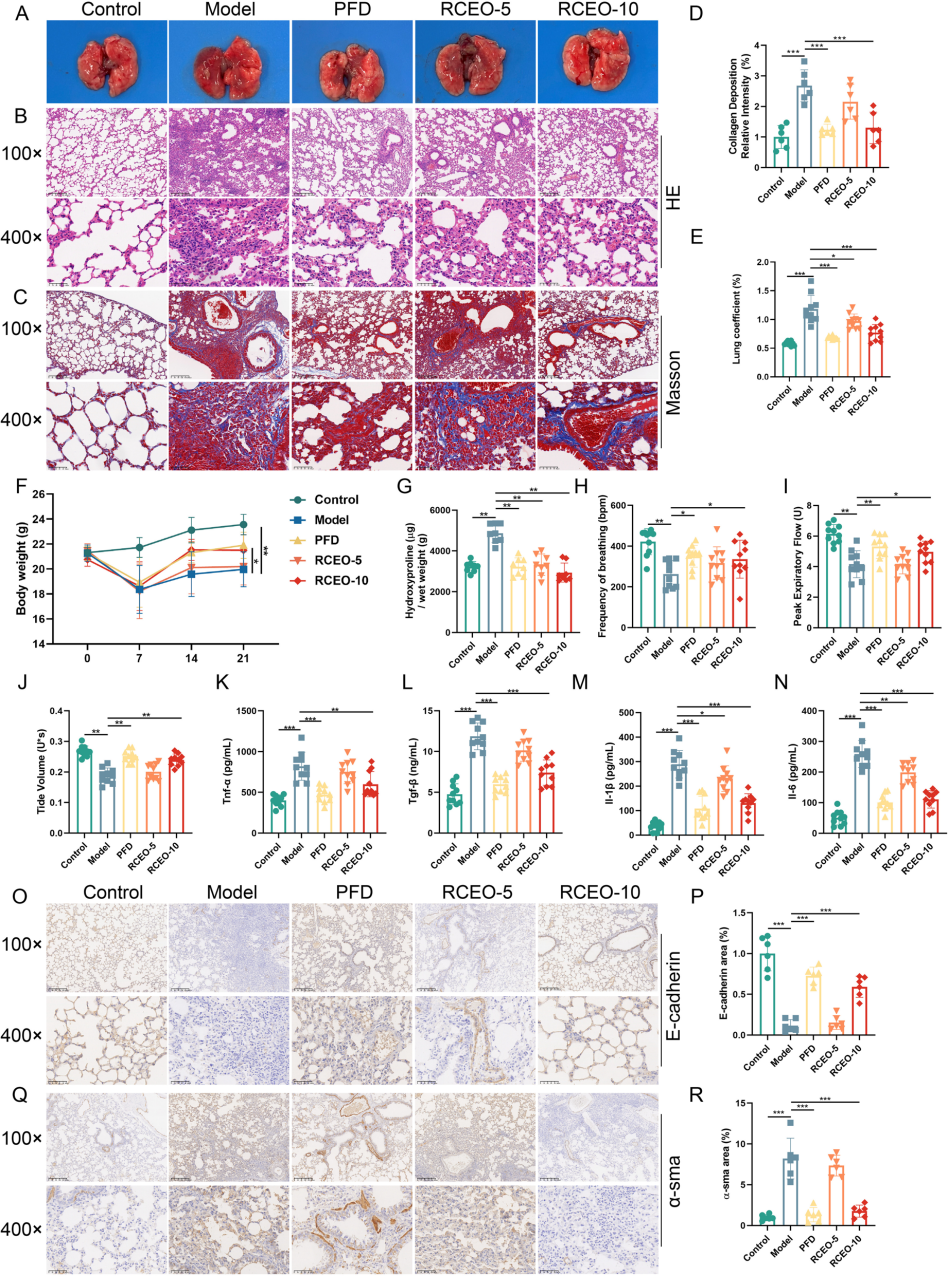
Effects of RCEO on pulmonary function in mice with pulmonary fibrosis. **A**–**C** Morphological changes in lung tissues were observed under light microscopy using HE staining and Masson’s trichrome staining (scale bars: 200 μm and 50 μm). **D** Optical density of collagen deposition was quantified using ImageJ following Masson’s staining (n = 6). **E**, **F** Changes in lung coefficient and body weight of mice during the experimental period (n = 8). **G** Hydroxyproline content in lung tissues (n = 8). **H**–**J** Alterations in pulmonary function parameters (respiratory frequency, peak expiratory flow, and tidal volume) (n = 8). **K**–**N** Serum levels of pulmonary fibrosis-related cytokines (Tnf-α, Tgf-β, Il-1β, and Il-6) (n = 10). (O-P) Immunohistochemical analysis of E-cadherin expression in lung tissues (n = 6; scale bars: 200 μm and 50 μm). **Q**-**R** Immunohistochemical analysis of α-sma expression in lung tissues (n = 6; scale bars: 200 μm and 50 μm). Data analysis was performed using one-way ANOVA and data were presented as mean ± SD. *P < 0.05, **P < 0.01

To assess the impact of RCEO on PF, a mouse model was established using intratracheal injection of BLM. RCEO inhalation therapy was administered from day 7 to day 21 following BLM injection, after which the mice were euthanized for analysis. Histopathological evaluation, employing HE and Masson staining, revealed significant inflammatory cell infiltration, collagen deposition, and alveolar structural distortion in the model group. In contrast, treatment with RCEO-10 significantly mitigated these pathological changes (Fig. 3A–D). A marked decrease in body weight was observed by day 7 post-BLM injection, with partial recovery by day 21. Additionally, lung coefficients and HYP content were significantly elevated. RCEO treatment effectively reversed these alterations, as evidenced by increased body weight and reduced lung coefficients and HYP levels (Fig. 3E–G). Pulmonary function tests further demonstrated that RCEO-10 ameliorated BLM-induced impairments in maximal expiratory flow, tidal volume, and respiratory frequency (Fig. 3H–J).

Given the pivotal roles of Tgf-β and α-sma as fibrotic markers, and the contribution of various inflammatory cytokines to the progression of fibrosis, an analysis was conducted on serum inflammatory factors and lung fibrosis markers [34]. PF mice demonstrated elevated serum levels of Tgf-β, Tnf-α, Il-1β, and Il-6, which were notably diminished following RCEO-10 inhalation (Fig. 3K–N). Considering the involvement of EMT and fibroblast-to-myofibroblast transformation in fibrosis, immunohistochemical staining was employed to evaluate the expression of E-cadherin (an epithelial marker) and α-sma (a fibrotic marker). The model group exhibited extensive α-sma-positive regions with limited E-cadherin expression within inflammatory cell clusters. In contrast, treatment with RCEO and PFD resulted in reduced inflammatory infiltration, suppression of α-sma, and restoration of E-cadherin expression. These findings indicate that RCEO significantly enhances pulmonary function in fibrotic mice (Fig. 3O–R).

### Effects of RCEO on macrophage-mediated PF progression

**Fig. 4:**
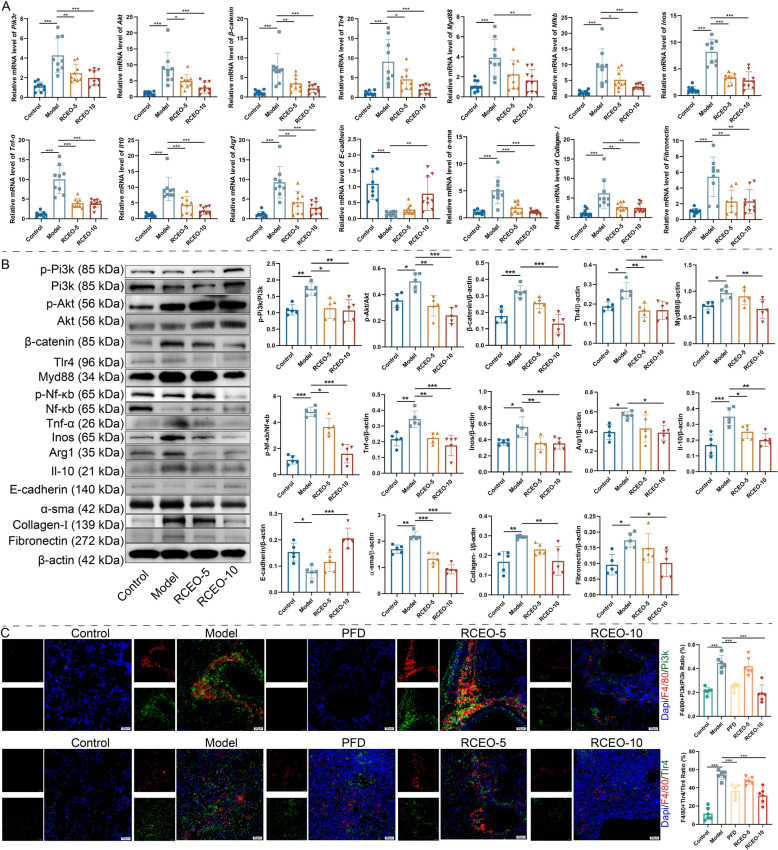
Effects of RCEO on macrophage-mediated pulmonary fibrosis progression. **A** Impact of RCEO on mRNA expression levels of *Pik3r, Akt, β-catenin, Tlr4, Myd88, Nfkb, Tnf-α, Inos, Il10, Arg1, E-cadherin, α-sma, Collagen-Ⅰ, Fibronectin* in lung tissues (n = 9). **B** Influence of RCEO on protein expression levels of Pi3k, Akt, β-catenin, Tlr4, Myd88, Nf-κb, Tnf-α, Inos, Il10, Arg1, E-cadherin, α-sma, Collagen-Ⅰ, Fibronectin in lung tissues (n = 5). **C** Effect of RCEO on immunofluorescence co-localization of F4/80 (a macrophage marker protein) with Pi3k and Tlr4 in lung tissues (n = 6). Scale bars: 50 μm. Data analysis was performed using one-way ANOVA and data were presented as mean ± SD. *P < 0.05, **P < 0.01

Macrophages are integral to the pathogenesis of PF. Research indicates that the inhibition of M2 macrophage polarization can effectively impede the progression of PF, while the accumulation of M1 macrophages is thought to significantly exacerbate fibrotic processes. The Pi3k/Akt/β-catenin and Tlr4/Myd88/Nf-κb signaling pathways are essential in modulating M2 and M1 macrophage polarization, respectively. This study employed qRT-PCR and WB to explore the involvement of these pathways in the potential ameliorative effects of RCEO on pulmonary function in fibrotic mice (Fig. 4A, B). The results demonstrated that mRNA and protein levels of fibrotic markers, including α-sma, Collagen-I, and Fibronectin, were significantly elevated in fibrotic lung tissues, while the epithelial marker E-cadherin was notably downregulated. Notably, inhalation of RCEO-10 effectively reversed these molecular alterations. The pathway proteins Pi3k, Akt, β-catenin, Tlr4, Myd88, and Nf-κb were notably upregulated, alongside an increased expression of macrophage markers inducible nitric oxide synthase (Inos) and Arg1, as well as inflammatory mediators Tnf-α and Il-10. Treatment with RCEO effectively suppressed these proteins, indicating that both M1 and M2 macrophage polarization contribute to fibrosis. RCEO appears to mitigate fibrosis by inhibiting macrophage polarization through these pathways. Immunofluorescence analysis further demonstrated elevated co-localization coefficients of the macrophage marker F4/80 with Pi3k and Tlr4 in fibrotic lungs, which were reduced following RCEO-10 treatment (Fig. 4C). These findings suggest that RCEO alleviates pulmonary fibrosis by inhibiting M2 polarization via the Pi3k/Akt/β-catenin pathway and M1 polarization via the Tlr4/Myd88/Nf-κb pathway, thereby reducing inflammation.

### RCEO’s impact on key cellular processes in PF

**Fig. 5:**
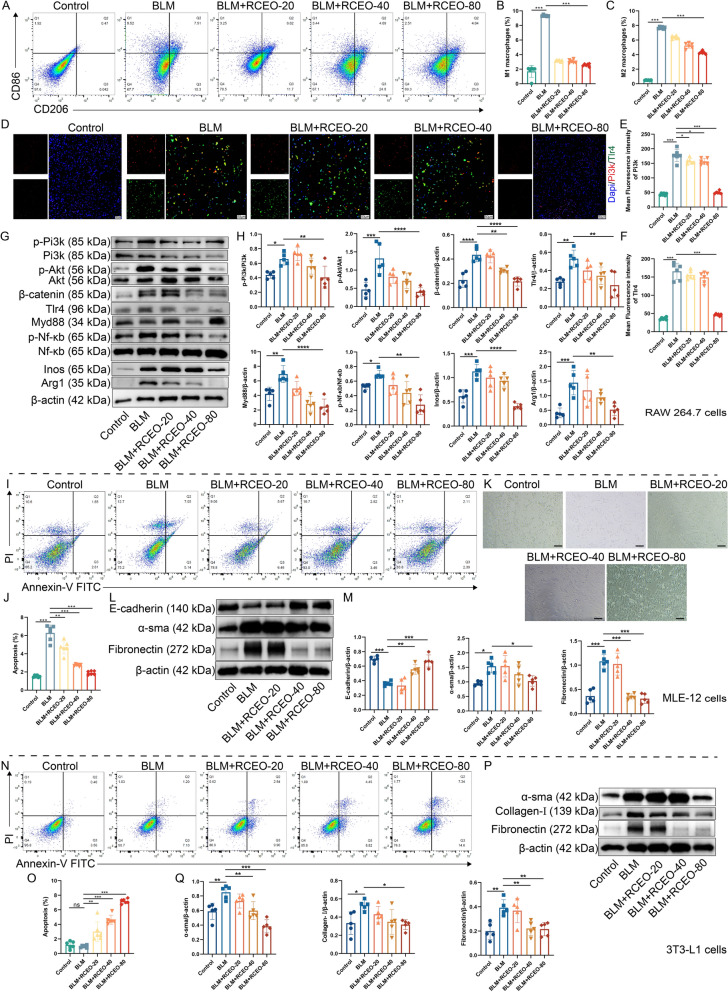
Effects of RCEO on multiple key cells in pulmonary fibrosis. **A**–**C** The impact of RCEO on RAW 264.7 polarization was assessed by flow cytometry (n = 6). **D**–**F** Effects of RCEO on the fluorescence intensity of Pi3k and Tlr4 in RAW 264.7 cells (n = 6). Scale bars: 50 μm. **G**, **H** Influence of RCEO on the expression of Pi3k, Akt, β-catenin, Tlr4, Myd88, Nf-κb, Inos, and Arg1 in RAW 264.7 cells (n = 5). **I**, **J** Effects of conditioned medium on the apoptosis rate of MLE-12 cells (n = 6). **K** Morphological changes in MLE-12 cells induced by conditioned medium. **L**, **M** Impact of conditioned medium on the expression of E-cadherin, α-sma, and Fibronectin in MLE-12 cells (n = 5). **N**, **O** Effects of conditioned medium on the apoptosis rate of 3T3-L1 cells (n = 6). **P**, **Q** Influence of conditioned medium on the expression of α-sma, Collagen-Ⅰ, and Fibronectin in 3T3-L1 cells (n = 5). Data analysis was performed using one-way ANOVA and data were presented as mean ± SD. *P < 0.05, **P < 0.01

Macrophage polarization, EMT, and FMT are critical processes in PF, with macrophage polarization playing a regulatory role in the latter two processes. In this study, we utilized a co-culture system to investigate the influence of RAW 264.7 macrophages on alveolar epithelial cells (MLE-12) and embryonic fibroblasts (3T3-L1). Induction with BLM significantly increased the populations of both M1 and M2 macrophages, an effect that was mitigated by RCEO-80 (Fig. 5A–C). Immunofluorescence and Western blot analyses demonstrated enhanced fluorescence intensity of Pi3k and Tlr4, as well as increased expression of pathway-related proteins in BLM-treated macrophages; these effects were reversed by RCEO-80 (Fig. 5D–H). In MLE-12 cells, flow cytometry, light microscopy, and Western blotting revealed that factors derived from BLM-activated macrophages promoted apoptosis and morphological changes (from cobblestone to spindle shape), along with decreased E-cadherin and increased α-sma and Fibronectin expression. RCEO-80 effectively counteracted these alterations, confirming its inhibitory effect on EMT (Fig. 5I–M). In 3T3-L1 cells, BLM treatment did not induce apoptosis but resulted in upregulation of α-sma, Collagen-I, and Fibronectin, all of which were suppressed by RCEO. These findings suggest that RCEO inhibits fibroblast activation, thereby reducing fibrosis (Fig. 5N–Q).

### Role of Pi3k/Akt/β-catenin and Tlr4/Myd88/Nf-κb pathways in macrophage-mediated regulation of MLE-12 and 3T3-L1 cells

**Fig. 6:**
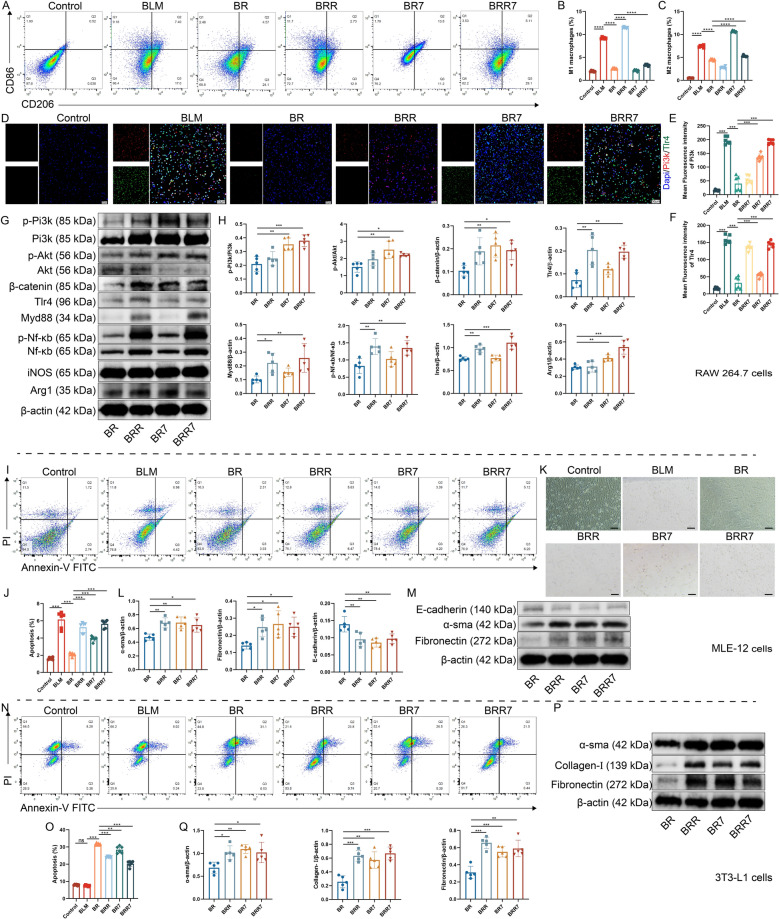
Effects of macrophage intervention via the Pi3k/Akt/β-catenin and Tlr4/Myd88/Nf-κb pathways on MLE-12 and 3T3-L1 cells. **A**–**C** Impact of RS09 and 740-Y-P on RAW 264.7 polarization (n = 6). **D**–**F** Effects of RS09 and 740-Y-P on intracellular Pi3k and Tlr4 fluorescence intensity in RAW 264.7 cells (n = 6; scale bars: 50 μm). **G**, **H** Influence of RS09 and 740-Y-P on the expression of Pi3k, Akt, β-catenin, Tlr4, Myd88, Nf-κb, Inos, and Arg1 in RAW 264.7 cells (n = 5). **I**, **J** Effect of conditioned medium on the apoptosis rate of MLE-12 cells (n = 6). **K** Morphological changes in MLE-12 cells induced by conditioned medium. **L**, **M** Impact of conditioned medium on the expression of E-cadherin, α-sma, and Fibronectin in MLE-12 cells (n = 5). **N, O** Effect of conditioned medium on the apoptosis rate of 3T3-L1 cells (n = 6). **P**, **Q** Influence of conditioned medium on the expression of α-sma, Collagen-I, and Fibronectin in 3T3-L1 cells (n = 5). Data analysis was performed using one-way ANOVA and data were presented as mean ± SD. *P < 0.05, **P < 0.01

To elucidate the roles of the pathways, macrophages were exposed to a Tlr4 agonist (RS 09) or a Pi3k agonist (740-Y-P), and their conditioned media were subsequently applied to MLE-12 and 3T3-L1 cells. The application of RCEO resulted in a reduction of M1/M2 polarization and a decrease in pathway protein expression. In contrast, RS 09 and 740-Y-P facilitated M1 and M2 polarization, respectively, demonstrating synergistic effects on specific proteins (Fig. 6. A-H). In MLE-12 co-cultures, both agonists intensified apoptosis and EMT markers, counteracting the beneficial effects of RCEO (Fig. 6I–M). In 3T3-L1 cells, the agonists decreased apoptosis and elevated fibrotic markers, such as α-sma, Collagen-I, and Fibronectin. Overall, RCEO mitigates fibrosis through the inhibition of dual pathways, thereby suppressing macrophage polarization and its subsequent fibrogenic effects (Fig. 6N–Q).

### Effects of the Pi3k/Akt/β-catenin and Tlr4/Myd88/Nf-κb pathways on pulmonary function in mice with PF

**Fig. 7:**
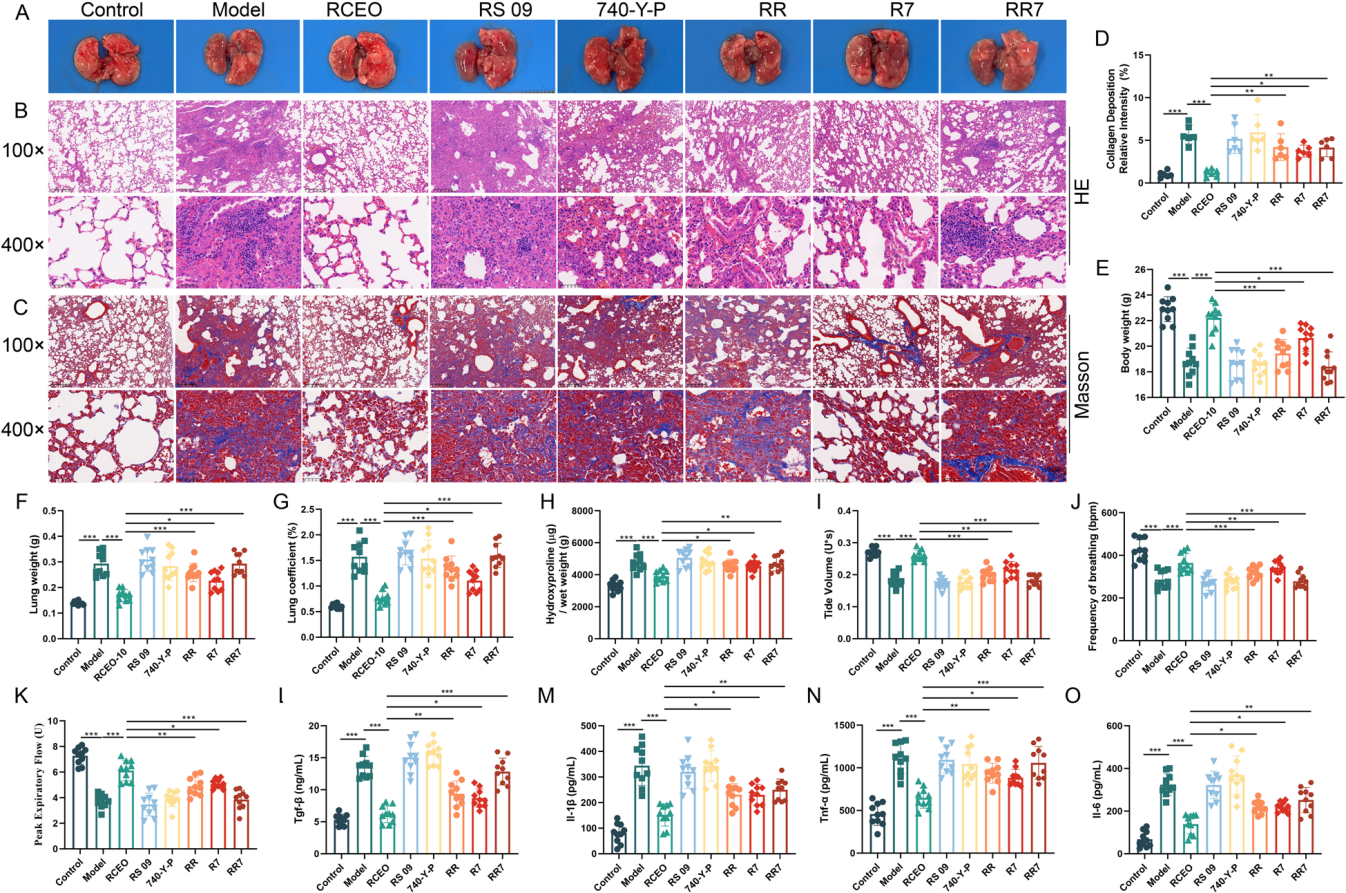
Effects of the Pi3k/Akt/β-catenin and Tlr4/Myd88/Nf-κb pathways on pulmonary function in mice with PF. **A**–**C** Morphological changes in lung tissues of mice treated with RS 09 and 740-Y-P were observed under light microscopy using HE staining and Masson’s staining (scale bars: 200 μm and 50 μm). **D** Effects of RS 09 and 740-Y-P on the optical density of collagen deposition after Masson’s staining (n = 6). **E**–**G** Effects of RS 09 and 740-Y-P on changes in body weight, lung weight, and lung coefficient (n = 8). **H** Effects of RS 09 and 740-Y-P on hydroxyproline content in lung tissues (n = 6). **I**–**K** Effects of RS 09 and 740-Y-P on pulmonary function parameters (respiratory rate, peak expiratory flow, and tidal volume) in mice (n = 8). **L**–**O** Effects of RS 09 and 740-Y-P on serum levels of pulmonary fibrosis-related cytokines (Tnf-α, Tgf-β, Il-1β, and Il-6) (n = 10). Data analysis was performed using one-way ANOVA and data were presented as mean ± SD. *P < 0.05, **P < 0.01

To assess the pivotal role of the Pi3k/Akt/β-catenin and Tlr4/Myd88/Nf-κb signaling pathways in the enhancement of pulmonary function by RCEO in mice with PF, we employed RS 09 and 740-Y-P to modulate the expression of these pathways and subsequently evaluated alterations in lung tissue function. The results, as evidenced by light microscopy, HE staining, and Masson staining, demonstrated that both RS 09 and 740-Y-P significantly augmented inflammatory cell infiltration, collagen deposition, and induced morphological changes in lung tissues (Fig. 7A–D). Furthermore, these pharmacological agents notably increased lung weight, lung coefficient, and HYP content, while concurrently reducing body weight in the RCEO-treated group, thereby effectively reversing pulmonary function parameters such as tidal volume, respiratory frequency, and peak expiratory flow (Fig. 7E–K). ELISA results revealed that RS 09 and 740-Y-P substantially elevated serum levels of Tgf-β, Il-1β, Tnf-α, and Il-6 (Fig. 7L–O). Collectively, these findings suggest that RCEO ameliorates pulmonary function in fibrotic mice by simultaneously inhibiting the Pi3k/Akt/β-catenin and Tlr4/Myd88/Nf-κb pathways. Notably, the combined use of RS 09 and 740-Y-P exerted a stronger reversal effect on RCEO’s efficacy than either drug alone, suggesting a synergistic interaction between these pathways in mediating RCEO’s therapeutic effects.

### RCEO suppresses macrophage polarization via the Pi3k/Akt/β-catenin and Tlr4/Myd88/Nf-κb pathways

**Fig. 8:**
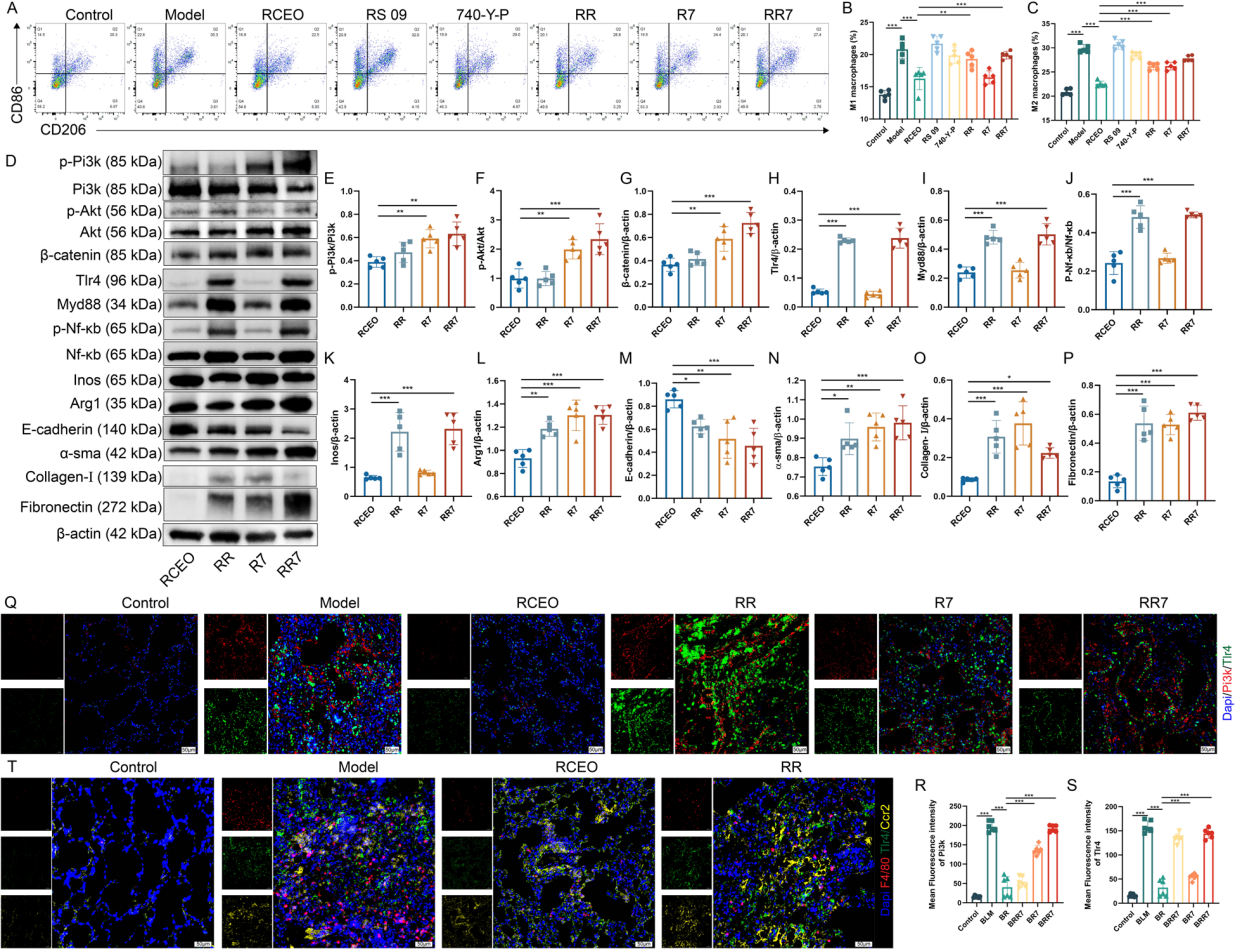
RCEO inhibits macrophage polarization via the Pi3k/Akt/β-catenin and Tlr4/Myd88/Nf-κb signaling pathways. **A**–**C** Effects of RS 09 and 740-Y-P on macrophage polarization in mouse lung tissues were assessed by flow cytometry (n = 6). **D**–**P** Effects of RS 09 and 740-Y-P on the expression of Pi3k, Akt, β-catenin, Tlr4, Myd88, Nf-κb, Tnf-α, Inos, Il-10, Arg1, E-cadherin, α-sma, Collagen I, and Fibronectin in lung tissues (n = 5). **Q**–**S** Effects of RS 09 and 740-Y-P on the fluorescence intensity of Pi3k and Tlr4 in mouse lung tissues (n = 6). Scale bars: 50 μm. **T** Co-localization of F4/80, Tlr4, and Ccr2 fluorescence in mouse lung tissues under the influence of Tlr4 and RS 09. Scale bars: 50 μm. Data analysis was performed using one-way ANOVA and data were presented as mean ± SD. *P < 0.05, **P < 0.01

Finally, to elucidate the roles of the Pi3k/Akt/β-catenin and Tlr4/Myd88/Nf-κb signaling pathways in the RCEO-mediated regulation of macrophage polarization and the progression of PF, we administered RS 09 and 740-Y-P and evaluated macrophage differentiation alongside pathway-related protein expression. RS 09 and 740-Y-P facilitated the polarization of M1 and M2 macrophages, respectively, with RS 09 also significantly enhancing M2 polarization (Fig. 8A–C). Western blot and immunofluorescence analyses demonstrated that both compounds upregulated the expression levels and fluorescence intensity of proteins associated with these pathways. Notably, RS 09 not only markedly increased the expression of the M1 marker iNOS but also augmented the expression of the M2 marker Arg-1, indicating its dual regulatory capacity (Fig. 8D–S). These findings imply that RS 09 modulates M2 macrophage polarization while exerting an influence on the Tlr4/Myd88/Nf-κb pathway. In addition, immunofluorescence analysis was conducted to investigate whether the effects of RS 09 on macrophages involved the suppression of recruitment and differentiation of circulating monocytes. The results indicated that RCEO inhalation therapy significantly decreased the colocalization of F4/80, Tlr4, and Ccr2 fluorescence in lung tissues. In contrast, RS 09 administration led to an increase in CCR2 fluorescence intensity and enhanced colocalization of F4/80, Tlr4, and Ccr2 (Fig. 8T). These findings suggest that RCEO ameliorates lung tissue pathology in fibrotic mice by inhibiting both M1 and M2 macrophage polarization through the Pi3k/Akt/β-catenin and Tlr4/Myd88/Nf-κb signaling pathways. Furthermore, RCEO's modulation of the Tlr4/Myd88/Nf-κb pathway not only suppresses M1 polarization but may also reduce M2 macrophage abundance by inhibiting the recruitment and activation of circulating monocytes.

## Discussion

Pulmonary fibrosis (PF) is a chronic interstitial lung disease marked by irreversible damage due to persistent injury and abnormal repair processes. The pathogenesis of PF involves various cell types, including immune cells such as macrophages and monocytes, as well as alveolar epithelial cells and fibroblasts. Alveolar macrophages play a crucial role in the recognition of foreign antigens, and their accumulation can influence alveolar epithelial cells and fibroblasts, thus facilitating the progression of PF. This study sought to elucidate the mechanisms through which RCEO confers protective effects on lung function in a murine model of PF. Our prior research demonstrated that RCEO improves lung function in murine models of pulmonary sarcoidosis by inhibiting the Tlr4/Myd88/Nf-κb signaling pathway-mediated inflammatory response. Utilizing bioinformatics and network pharmacology analyses, we further elucidated that the therapeutic effects of RCEO on PF may be linked to the Pi3k/Akt/β-catenin and Tlr4/Myd88/Nf-κb pathways. To screen for the optimal drug concentration, RAW 264.7 cells, MLE-12 cells, and 3T3-L1 cells were co-incubated with RCEO for 6, 12, and 24 h, and changes in cell viability were assessed. The screening strategy was based on the premise that RCEO would not significantly affect the viability of RAW 264.7 cells and MLE-12 cells but would inhibit the viability of 3T3-L1 cells, thereby reducing fibroblast activation and extracellular matrix release. The results indicated that the optimal concentrations for subsequent cellular experiments were RCEO-20, RCEO-40, and RCEO-80. Subsequent in vivo and ex vivo experiments corroborated that RCEO impedes the polarization of M1 and M2 macrophages (i.e., macrophage depletion), inhibits EMT in alveolar epithelial cells and FMT, and diminishes the expression of ECM marker proteins, including α-sma, Collagen-I, and Fibronectin. These findings collectively suggest that RCEO contributes to the amelioration of lung function in fibrotic mice.

The hallmark of PF is aberrant tissue repair, which is characterized by excessive ECM deposition predominantly produced by fibroblasts and myofibroblasts. Under pathological conditions, FMT plays a crucial role in ECM production, while type II epithelial cells undergoing EMT also significantly contribute to ECM accumulation [35–37]. Research has demonstrated that inhibiting mesenchymal cell activity and obstructing ECM expression and release can effectively alleviate PF. In this study, we observed increased mRNA and protein levels of ECM components, including α-sma, Collagen-I, and Fibronectin, in the lung tissues of fibrotic mice, along with a marked reduction in the epithelial marker E-cadherin, as confirmed by immunohistochemical staining. These findings indicate a substantial increase in ECM deposition and FMT in fibrotic lungs. Furthermore, the decreased expression of E-cadherin suggests the occurrence of EMT during the progression of fibrosis. Notably, treatment with RCEO significantly reversed these pathological alterations, demonstrating its efficacy in suppressing EMT and FMT, thereby enhancing lung function in fibrotic mice.

Macrophages are integral to the pathogenesis of PF [38, 39]. Upon encountering exogenous pathogens, macrophages differentiate into the M1 phenotype and release proinflammatory cytokines to combat the pathogens. However, sustained stimuli can activate tissue repair mechanisms, leading to macrophage polarization towards the M2 phenotype, which secretes profibrotic cytokines such as TGF-β, thereby exacerbating fibrotic processes. Both M1 and M2 macrophages are critically involved in distinct stages of fibrosis development. Research indicates that inhibiting M2 polarization can reduce TGF-β secretion and ameliorate fibrosis [40, 41], while enhancing M1 polarization may decrease the abundance of M2 macrophages, thus attenuating fibrotic progression. Conversely, some studies suggest that suppressing M1 polarization also yields therapeutic benefits [42, 43]. M1 macrophages are principal producers of chemokines such as CCL2 and CCL7, which recruit circulating monocytes to injured pulmonary tissue via the CCL2-CCR2 axis. Under pathological conditions, monocytes constitute a major source of lung macrophages [44, 45]. Recent evidence indicates that lung macrophages can be categorized not only based on their functional characteristics (M1, classically activated; M2, alternatively activated) but also according to their origin: alveolar macrophages, interstitial macrophages, and monocyte-derived macrophages. Alveolar and interstitial macrophages, collectively known as tissue-resident macrophages, have the capacity to polarize into either M1 or M2 subsets during fibrotic processes [46]. Conversely, monocyte-derived macrophages tend to initially assume an M1 phenotype before transitioning to an M2 phenotype. Consequently, the specific roles of these distinct macrophage subsets in fibrosis remain a subject of ongoing debate. Recent studies suggest that both M1 and M2 macrophages are present in fibrotic lungs, challenging the traditional view that M1 polarization is confined to the inflammatory phase while M2 polarization is restricted to the fibrotic phase [47]. The concurrent inhibition of M1 and M2 macrophage polarization, achieved through macrophage ablation, significantly enhances pulmonary function in murine models of fibrosis. Flow cytometry analysis demonstrated increased populations of both M1 and M2 macrophages in fibrotic lung tissue, indicating their joint contribution to the fibrotic process. Treatment with RCEO resulted in a reduction of these macrophage subsets and a decrease in the expression of M1 and M2 markers, specifically iNOS and Arg1. This intervention subsequently led to a reduction in the release of proinflammatory and profibrotic cytokines, thereby mitigating pulmonary fibrosis.

Regulation of Macrophage Recruitment and Activation in Pulmonary Fibrosis: Mechanistic Insights into RCEO-mediated Modulation of Tlr4/Myd88/Nf-κb and Pi3k/Akt/β-catenin Pathways. The recruitment and activation of macrophages in vivo are orchestrated by multiple signaling pathways, with the Tlr4/Myd88/Nf-κb axis being the primary pathway that promotes M1 macrophage polarization [48]. The transmembrane receptor Tlr4 detects exogenous stimuli and recruits myeloid differentiation primary response 88 (Myd88), a pivotal adaptor protein in Tlr4 signaling, thereby facilitating the phosphorylation of nuclear factor kappa-light-chain-enhancer of activated B cells (Nf-κb), a crucial transcription factor within macrophages. Upon phosphorylation, Nf-κb translocates to the nucleus, where it binds to specific DNA sequences to induce the release of pro-inflammatory cytokines, thereby initiating inflammatory cascades and M1 polarization. Research indicates that FCP mitigates pulmonary fibrosis by inhibiting Tlr4/Nf-κb-mediated M1 macrophage inflammatory responses [49]. In contrast, the Pi3k/Akt/β-catenin pathway is recognized as the classical route for M2 macrophage polarization [50]. Interleukin-4 (IL-4), a prototypical factor that induces M2 macrophage polarization, initiates a sequential phosphorylation process involving phosphoinositide 3-kinase (Pi3k) and protein kinase B (Akt). This phosphorylation cascade subsequently results in the dephosphorylation of glycogen synthase kinase 3 beta (GSK-3β), which stabilizes β-catenin and facilitates its translocation to the nucleus. This signaling pathway promotes the release of anti-inflammatory and pro-fibrotic cytokines, ultimately leading to M2 macrophage polarization. Recent investigations have demonstrated that ISE II exhibits significant anti-fibrotic properties by inhibiting the Pi3k/Akt/β-catenin-mediated activation of M2 macrophages [15]. The Pi3k/Akt signaling pathway exerts a regulatory role on the Tlr4/Myd88 signaling pathway and the subsequent inflammatory response. Activation of the Pi3k/Akt pathway can stimulate the Nf-κb signaling pathway and the expression of related inflammatory factors. Pi3k can also interact with Myd88, and the interaction between the Pi3k p85 subunit and MyD88 is considered a key factor for Akt phosphorylation. Blocking the Tlr4/MyD88/NF-κB and Pi3k/Akt signaling pathways can ameliorate oxidative stress and inflammation in acute liver injury [51]. The Pi3k/Akt and Tlr4/Myd88 signaling pathways are also critical for immune regulation [52]. In diseases such as PF, asthma and myocardial infarction, pharmacological agents can achieve therapeutic effects through dual modulation of the Pi3k/Akt and Tlr4/Myd88 signaling pathways [16, 53, 54]. In the present study, bioinformatics and network pharmacology analyses have preliminarily identified the potential anti-fibrotic mechanisms of RCEO, implicating both the Pi3k/Akt/β-catenin and Toll-like receptor 4 (Tlr4)/Myd88/nuclear factor kappa-light-chain-enhancer of activated B cells (Nf-κb) pathways. Polymerase Chain Reaction (PCR), Western blot (WB), and immunofluorescence assays revealed significantly increased mRNA and protein expression levels of these pathways in the lung tissues of fibrotic mice. In contrast, treatment with RCEO-10 markedly suppressed these expressions and decreased the levels of M1/M2 macrophage marker proteins. These results indicate that RCEO ameliorates pulmonary fibrosis by inhibiting macrophage polarization. To ascertain the essential role of these pathways, the Tlr4 agonist RS 09 and the Pi3k agonist 740-Y-P were utilized to counteract the effects of RCEO. Both agonists selectively activated their respective pathway proteins and diminished the therapeutic efficacy of RCEO. Importantly, RS 09 and 740-Y-P demonstrated synergistic effects, corroborating that RCEO alleviates pulmonary fibrosis primarily through the dual inhibition of the Pi3k/Akt/β-catenin and Tlr4/Myd88/Nf-κb pathways, thereby preventing macrophage polarization.

During the investigation into the potential mechanisms by which RCEO ameliorates PF, it was notably observed that RS 09 not only significantly increased the population of M1 macrophages but also induced the polarization of M2 macrophages and upregulated the expression of macrophage-associated proteins. Previous studies have demonstrated that, under pathological conditions, the recruitment of circulating monocytes is primarily dependent on the interaction between the surface receptor Ccr2 and its ligands, such as Ccl2, Ccl7, and Ccl8. Upon recruitment to the site of injury, circulating monocytes rapidly differentiate into macrophages and perform their functional roles. In the context of PF, M1 macrophages, rather than M2 macrophages, serve as the primary source of key chemokines, including Ccl2, Ccl7, and Ccl8. In this study, we posited that RS 09 plays a regulatory role in macrophages by promoting M1 macrophage differentiation, which in turn enhances the release of specific chemokines and facilitates the recruitment of circulating monocytes to injured lung tissue. Immunofluorescence analysis was conducted to evaluate the colocalization of F4/80 (a macrophage marker), Ccr2 (a monocyte marker), and Tlr4. The detection of Ccl2 concentration in the supernatant of RAW 264.7 cells revealed that RCEO significantly inhibited the release of Ccl2 from RAW 264.7 cells, while RS 09 markedly reversed the effect observed in the RCEO group. The findings indicated that RCEO reduced the colocalization of these markers, whereas RS 09 increased their colocalization. Overall, these results suggest that RCEO alleviates pulmonary fibrosis by inhibiting M1 macrophage polarization and obstructing the recruitment and activation of circulating monocytes through the Tlr4/Myd88/Nf-κb signaling pathway, while simultaneously reducing M2 macrophage polarization.

The principal findings of this study indicate that RCEO exhibits anti-PF effects by inhibiting macrophage polarization through the Pi3k/Akt/β-catenin and Tlr4/Myd88/Nf-κb signaling pathways. Furthermore, it was observed that the inhalation route of RCEO administration facilitates the delivery of a majority of its components into lung tissue, with 17 out of 24 major components detected, accounting for over 70%. These results propose that inhalation could serve as a viable drug delivery strategy for pulmonary diseases, enhancing effective absorption of drug molecules while minimizing systemic side effects. Despite the significant pharmacological advantages of inhalation administration in delivering complex essential oil components to lung tissues, several key challenges must be addressed to translate this approach into clinically viable inhalation formulations. First, the physical and chemical stability of the formulation is a prerequisite for successful translation. Future research should focus on screening suitable excipient systems to ensure the chemical integrity of active ingredients during storage and use. Second, pulmonary deposition efficiency is critical for effective delivery. The efficacy of inhalation formulations is highly dependent on their aerodynamic particle size. For optimal pulmonary delivery, the aerodynamic diameter should be within the range of 1–5 μm to avoid oropharyngeal deposition and achieve deep lung tissue distribution. Subsequent studies should utilize devices such as the Next Generation Impactor (NGI) to evaluate aerosol characteristics under different nebulization conditions and optimize formulation parameters to ensure efficient deposition of RCEO components in the alveolar target region. Finally, clinical feasibility and safety are ultimate considerations for translation. Extrapolating from small animal inhalation studies to humans requires complex interspecies dose conversion and consideration of the more complex respiratory physiology in humans. Moreover, the potential irritant effects on the respiratory mucosa or systemic toxicity associated with long-term inhalation of complex essential oils must be systematically evaluated through standardized toxicological studies. Additionally, ex vivo experiments demonstrated that RCEO influences apoptosis and related protein expression in MLE-12 and 3T3-L1 cells through the regulation of macrophages. These findings suggest that macrophage-mediated modulation may represent a promising therapeutic strategy for mitigating PF. This study, however, is subject to several limitations. Firstly, the anti-fibrotic effects of RCEO may be attributed to multiple components, including Germacrone, Hydroxyvalerenic Acid, and Velleral, which demonstrate dual-targeting effects on both pathways. This necessitates further investigation into their specific roles in the amelioration of PF. A concentration gradient was established for three potential active components, followed by cell safety and efficacy assays to evaluate their safety and efficacy from an ex vivo perspective. The screened active components were then combined to investigate potential synergistic effects among different components. Furthermore, animal experiments were conducted to assess their safety, efficacy, and synergistic interactions in order to identify the precise active pharmaceutical ingredients. Secondly, while pathway-specific agonists (RS 09 and 740-Y-P) were utilized to elucidate the involvement of these pathways in RCEO-mediated fibrosis alleviation, more precise genetic editing techniques are required for comprehensive mechanistic validation. Furthermore, although the pivotal roles of Pi3k and Tlr4 in PF have been established, the regulatory mechanisms of their active subunits remain unexplored. While this study suggests that RCEO may mitigate PF by inhibiting the recruitment of circulating monocytes to injured lung tissue via the Tlr4/Myd88/Nf-κb pathway, the underlying mechanisms of intervention warrant further investigation.

## Conclusion

In summary, RCEO ameliorates lung function in PF mice by inhibiting the Pi3k/Akt/β-catenin and Tlr4/Myd88/Nf-κb signaling pathways, thereby blocking macrophage polarization in lung tissue, reducing the release of pro-inflammatory and pro-fibrotic factors, and decreasing extracellular matrix deposition. Inhibition of macrophage polarization in lung tissue may represent a potential therapeutic strategy for PF.

## Supplementary Information

Additional file 1.

Additional file 2.

## Data Availability

No datasets were generated or analysed during the current study.

## Abbreviations

Arg-1: Arginase-1
BLM: Bleomycin
CETSA: Cellular Thermal Shift Assay
DMSO: Dimethyl sulfoxide
ECM: Extracellular matrix
EMT: Epithelial-mesenchymal transition
FMT: Fibroblast-to-myofibroblast transition
GC–MS: Gas Chromatography-Mass Spectrometry
GEO: Gene Expression Omnibus
GO: Gene Ontology
HYP: Hydroxyproline
Inos: Inducible nitric oxide synthase
KEGG: Kyoto Encyclopedia of Genes and Genomes
PEF: Peak expiratory flow
PF: Pulmonary fibrosis
PFD: Pirfenidone
RCEO: Essential oil from *Radix Curcumae*
TGF-β: Transforming growth factor-beta
TV: Tidal volume
α-sma: α-Smooth muscle actin
