# Supplementary material for: Exploring the role of RCEO in macrophage-mediated modulation of pulmonary fibrosis
Source: Chin Med. 2026 May 29;21:153. doi: 10.1186/s13020-026-01398-w (PMC13220479; doi:10.1186/s13020-026-01398-w)
Supplement: Supplementary file 1 — Additional file 1. [file 13020_2026_1398_MOESM1_ESM.pdf]

河南中医药大学实验动物福利伦理审查表

Application Format for Ethical Approval for Research Involving Animals

Henan University of Traditional Chinese Medicine

申请日期：2024 年 05 月 14 日　　　　　　　　批准文号：IACUC-202405027
Appl.Date：2024 Y　05 M　14　D　　　　　　IACUC Issue No. IACUC-202405027

课题名称及编号<br>Program and No.	郁金精油对肺纤维化小鼠的作用机制研究	课题来源<br>Sponsor	自拟
课题负责人<br>Name of Principal Investigator	苗明三	科室<br>Department	中医药科学院
动物试验负责人<br>Contact Person	茆志国	电话和信箱<br>Contact Tel.No.and Email	mzghefei@163.com
课题实施动物实验的人数<br>Number of Implement	2	经专业培训的人数<br>Number of certificate	2

参与动物实验操作人员姓名、相关专业证书编号，经验、培训、资质和能力的描述
Name and certificate number, Description of experience/training/competency of the individuals carring out the research.

茆志国　参加了学校动物实验中心准入培训班，经考试合格。获得了实验动物从业人员的相关资质和技能，了解了实验动物福利伦理有关法律、法规，了解了善待实验动物的知识。经过动物中心培训熟练掌握了正确的实验操作技术。

刘莹　参加了学校动物实验中心准入培训班，经考试合格。获得了实验动物从业人员的相关资质和技能，了解了实验动物福利伦理有关法律、法规，了解了善待实验动物的知识。经过动物中心培训熟练掌握了正确的实验操作技术。

动物实验设施许可证编号<br>Name and certificate number of the facility	SYXK（豫）2021-0015	特殊实验设施许可证编号<br>Name and certificate number of the facility	无<br>None
现有动物实验设施条件是否与拟开展动物实验的规范性要求相匹配的描述<br>Conformity of facility condition and proposed to carry out experiment requirement<br>现有动物实验设施条件与拟开展动物实验的规范性要求相匹配。本实验拟于河南中医药大学实验动物中心开展，能够为实验动物提供符合动物等级要求，且清洁、舒适、安全的生活环境。本动物实验拟于严格控制人流、物流和空气流的屏障环境中饲养。设施的选址、建筑卫生、环境等级等均符合动物实验要求。并且本动物实验完成所需的实验器材相对简单，实验动物中心能够提供相应的实验条件。			
拟实验时间：2024 年 05 月 30 日 至 2024 年 09 月 30 日<br>Experimental period：2024 Y 05 M 30 D to 2024 Y 09 M 30 D			

动物实验项目的目的、必要性、意义和如何设计达成研究目标的

Experimental objective, necessity and significance and how the program has been designed to achieve the objectives of the research.

目的：探讨郁金精油是否可改善肺纤维化（PF）小鼠的肺组织结构和功能，以及其药效作用的潜在机制是否与巨噬细胞的的募集活化有关。

必要性：肺纤维化是一种慢性不可逆的间质性肺部疾病，主要以细胞外基质过度沉积和异常修复为特征。目前 FDA 批准用于治疗肺纤维化一线药物包括尼达尼布和吡啡尼酮，这两种药物可有效减缓疾病发展，提高患者的生存期和生活质量，但难以治愈肺纤维化。因此，迫切需要探索肺纤维化的潜在发病机制和新的治疗方法。本项目前期实验研究显示，郁金精油对慢性肺组织疾病具有较好的保护作用。因此，需要通过动物实验研究，进一步明确郁金精油对 PF 小鼠模型的治疗效果和潜在作用机制。

意义：探讨郁金精油通过调节巨噬细胞的募集活化，减少肺组织中细胞外基质聚集并改善 PF 小鼠肺组织结构和功能，将为临床治疗 PF 提供理论依据。

达成研究目标的方法：本实验考察郁金精油对博来霉素诱导的 PF 小鼠模型的治疗作用，雄性小鼠 C57BL/6J 共 50 只，购回后适应性一周。实验分组为空白组 10 只，博来霉素组 10 只，博来霉素加吡啡尼酮组 10 只，博来霉素加郁金精油高剂量组 10 只，博来霉素加郁金精油低剂量组 10 只。采用气管注射博来霉素制备 PF 小鼠模型。除空白组外，其余各组小鼠气管注射给予博来霉素，单次气管注射造模，模型持续 3 周。博来霉素加郁金精油高剂量组、博来霉素加郁金精油低剂量组以及博来霉素加吡啡尼酮组分别于气管注射博来霉素 1 周后开始给药，博来霉素加郁金精油高剂量组吸嗅给予剂量 0.1 mL/kg 郁金精油，博来霉素加郁金精油低剂量组吸嗅给予剂量 0.05 mL/kg 郁金精油，博来霉素加吡啡尼酮组灌胃给予 300 mg/kg 吡啡尼酮，其余各组给予等量橄榄油。待造模 3 周后处死小鼠，收集肺组织，检测肺组织病理和相关蛋白表达情况等。

<table>
<tr><td rowspan="4">拟使用动物信息 Animal to be used</td><td>动物来源
Animal origin
许可证编号
Certificate number</td><td colspan="2">山东朋悦实验动物繁育有限公司
许可证号:SCXK (鲁)20220006</td><td>质量合格证
Certification of fitness</td><td>☑有
☐无</td></tr>
<tr><td colspan="3">品种/品系
Breed/strain
☐大鼠 rat: ____________ ☑小鼠 mice: C57BL/6J 小鼠
☐裸鼠 nude mice: _______ ☐兔 rabbit: ________
☐犬 dog: _____________☐灵长类 primary animal: _____
☐转基因动物 genetically modified animal: ______________
☐其他（具体说明）other: ____________________</td><td>等级
Grade</td><td>☐普通
☑SPF
☐GF
☐其他</td></tr>
<tr><td colspan="2">数量只（♀ 0 只 ♂ 50 只 ）
Number（♀ 0 ♂ 50 ）</td><td>体重 20-22 g
Weight 20-22 g</td><td colspan="2">☐日龄 D
Age day
☑周龄 6-8 W
Age week
☐月龄 M
Age moth</td></tr>
<tr><td colspan="5">选择实验动物种类和数量的原因
Reasons for the choice of special and numbers of animals to be used.
种类：本实验选择 C57BL/6J 小鼠是因为 C57BL/6J 小鼠对博来霉素较为敏感，能够在单次气管注射博来霉素后表现出肺部的病理改变，且具有与人类相似的 PF 肺部病理特点。故本实验选择 C57BL/6J 小鼠作为实验对象。

数量：本实验室共分为正常对照组、博来霉素组、博来霉素加吡啡尼酮组、博来霉素加郁金精油高剂量组、博来霉素加郁金精油低剂量组共五组。为各组具有统计学意义，并排除个体差异，故每组 C57BL/6J 小鼠的数量定为 10 只。</td></tr>
</table>

拟开展动物实验的详细信息 Detailed information of the experiments on animals	详细列出对动物可能造成的所有可预期的伤害，包括动物运输、每个实验方案动物饲养方式、实验操作步骤中等可能产生伤害或不适的细节及拟采取的防控措施<br>Description of the overall harms expected to be experienced by the animals-including details of the likely adverse effects of each protocol，cage breeding and the steps which will be taken to control these adverse effects。<br>在运输过程中，实验动物可能会受到天气温度、湿度等各种因素的影响，故本实验选择在相对气温比较稳定的时期开展，由实验动物生产单位负责运输，选用专用运输车，通过快速便捷的陆路运输方式完成动物的运输，尽量避免实验动物在运输途中可能受到的影响及伤害。<br>在饲养过程中，实验动物可能会因为个体空间、食物与水等资源的问题而出现相互伤害的现象。所以本实验采用每 5 只/笼进行，能够满足实验动物所占笼具最小面积的国家标准。此外，可为动物准备一些玩具或娱乐设施等丰盈实验动物的生活环境。<br>小鼠之间存在个体差异，在麻醉时可能会出现麻醉过量而导致小鼠死亡的情况发生。因此每次实验前对实验小鼠进行体重称量，严格按照体重和麻醉剂量等进行麻醉。<br>在气管注射博来霉素过程中，实验小鼠可能由于麻醉和气管注射手术操作导致出现不适，实验过程中确保小鼠在加热操作台上进行，术后维持小鼠体温，如发现实验动物状态较差时将暂停手术，待动物回复正常后继续进行手术操作。<br>尽量减少动物在实验过程中的痛苦和惊恐，我们严格按照操作规程的要求，对动物轻拿轻放，实验中注意对动物进行抚慰，待动物安静后方进行吸嗅和灌胃给药操作。

	主要观察指标<br>Main observation target<br>1. 小鼠的体重（the weight of the mice）：每周测量一次；<br>2. 肺组织病理学分析和 Masson 染色检测（Pathological analysis and Masson staining of lung tissue）：实验结束后，小鼠腹腔注射巴比妥钠处死，采集小鼠左肺组织采用 10%甲醛固定，脱水后石蜡包埋。随后，蜡块切片，进行 HE 和 Masson 染色；<br>3. 肺组织巨噬细胞及通路等相关蛋白水平检测（Detection of protein levels related to macrophages and pathways in lung tissue）：实验结束后，小鼠腹腔注射巴比妥钠处死，采集小鼠右肺组织。采用 western blot 技术检测巨噬细胞及通路等相关蛋白表达水平。
拟开展动物实验的详细信息 Detailed information of the experiments on animals	仁慈终点或实验终结的指标<br>Humance endpoint or experimental terminative indicator<br>本实验过程中可能会导致小鼠消瘦、体重减轻、食量减少等症状，所以本实验的仁慈终点或实验终结的指标为：<br>1. 体重减轻：体重减轻达动物原体重的 20%-25%，或动物出现恶病质或消耗性症状；<br>2. 食欲丧失：完全食欲丧失达 24 h 或食欲不佳（低于正常食量的 50%）达 3 天；<br>3. 虚弱(无法进食或饮水):动物在没有麻醉或镇静的状态下，无法进食或饮水，长达 24 小时无法站立或极度勉强才可站立时；<br>4. 垂死/濒死:动物在没有麻醉或镇静的状态下，表现精神抑郁伴随体温过低(低于 37℃)时；<br>5. 皮肤:无法治愈之伤口、重复性自残或二级以上之保温垫烫伤。

<table>
<tr><td></td><td>动物处死方法
Death conduct
腹腔注射巴比妥钠类药物注射液，剂量 100 mg/kg，麻醉过量致死。</td></tr>
<tr><td></td><td>非处死动物的处置方法
Not for the death of the animal disposition
☑继续使用
Continue to use
☐保存的机构
Save in the agency
☐放生野外
Release to the wild
☐其他，详细说明
Others，detailed description</td></tr>
<tr><td>拟开展动物实验的详细信息 Detailed information of the experiments on animals</td><td>动物替代、减少动物用量、降低动物痛苦伤害的主要措施
Major measure for 3Rs
由于实验的最终目的是评价郁金精油对 PF 小鼠模型的治疗效果，由于目前其机制、原理还不清楚，需要借助动物实验阐明其药效作用机制。该项目目前不可以用计算机模拟、细胞培养等非生命方法替代，本实验选用了低等动物小白鼠替代了高等动物。
小鼠是适合我们试验最低等最经济合理的动物。
我们前期经过和有经验的老师讨论研究，优化了本次动物实验的方案，并且已经进行了必要的优化，采用了最少的实验动物来做实验。最后所选取每组 10 只 C57BL/6J 小鼠是最适合我们实验方案的，可以节省大量的资源和生命。
在使用动物进行有关实验时，我们尽量减少非人道程序对动物的影响范围和程度，在实验过程中尽量采用物理、化学及体外检测等非夺取动物生命的方法。在实验开始前，充分了解实验动物的生理生态习性，熟练掌握抓取、注射、取样、测量等操作技巧，对动物身体的强制性限制减少到最低程度。
实验过程中，确保动物在麻醉、镇痛、镇静、或其他适当手段下进行实验，避免、减少或减轻给动物造成的伤害和痛苦，以保证动物健康和康乐，保证动物实验结果的可靠性和采用提高实验动物福利的科学方法。
在实验过程中，处理动物有适当的时间间隔，并减少必要的试验处理，尽量不增加动物的痛苦。同时，我们在实验过程中，严格按照操作规程的要求饲喂动物，给予动物舒适良好的生存环境，让动物能够安全舒适的生活和休息。
实验过程中，实验动物如果出现一些较大伤害，造成动物濒死或影响其正常生存，达到仁慈终点时，我们便即刻对动物实施安乐死。
在对实验动物进行解剖时，将进行有效麻醉。处死实验动物时，将按照人道主义原则实施安死术。处死现场，不宜有其他动物在场。确认动物死亡后，方可妥善处置尸体。</td></tr>
</table>

是否使用有毒（害）物质（感染、放射、化学毒、其他）

Poisonous（harmful）material（infection，radiate，chemical poison and other）being used

☑否 no

☐是 yes

说明：

Declare

无

利害分析的小结，说明为何预期的利益多于害处？

A summary of the harm-benefit analysis - why the expected benefits might be considered to outweigh the predicated harms?

肺纤维化是一种慢性不可逆的间质性肺部疾病，主要表现为肺组织功能严重降低和呼吸困难，中位生存期不超过 5 年。目前 FDA 批准用于治疗肺纤维化一线药物包括尼达尼布和吡啡尼酮，这两种药物可有效减缓疾病发展，提高患者的生存期和生活质量，但难以治愈肺纤维化。因此，迫切需要探索肺纤维化的潜在发病机制和新的治疗方法。课题组前期研究证实郁金精油在细胞水平对 PF 具有一定治疗作用，但对其在动物层面的药效和作用机制研究尚属空白。本实验具有创新性。

技术创新能力：揭示郁金精油治疗 PF 的药效作用机制。

经济指标：研发改善 PF 临床症状的新药物，为临床广大患者节约大量的资金，并缩短住院周期。

社会效益：1、为郁金精油治疗 PF 提供科学依据。

2、可以为临床广大患者节约大量的资金，并缩短住院周期。

相关的补充说明或辅助证明文件<br>Supplementary instruction or any auxiliary documents for investigate<br><br>None
信息公开和保密要求：说明哪些信息需要保密，哪些信息可以公开<br>Declaration for the information disclosure and confidentiality requirements，declaring the information need to be kept secret，the information can be disclosed。<br><br>无保密信息<br>None
对伦理委员有无回避要求<br>Claiming jurors for being debarb.<br><br>无<br>None

声明：1.我将自觉遵守实验动物福利伦理相关法规和各项规定，同意接受伦理委员会和实验动物室管理者的监督与检查。<br>2.本人保证本申请表中所填内容真实、详尽和易懂。<br>Declaration：1. I will abide by the law and regulation stipulation，and accept the supervision and inspection by the committee and laboratory animal department。<br>2. The information I have given is accurate，detail and comprehensive。<br><br>声明人：课题负责人签（章）<br>Declarant：Signature （stamp）of PI<br><br>动物实验负责人签（章）<br>Signature （stamp）of Director of animal experiment<br>2024 年 05 月 14 日<br>2024 Y 05 M 14 D
主管兽医师意见<br>Opinion of Veterinary of institution<br><br>同意<br><br>主管兽医师签（章）：<br>Signature （stamp）of Veterinary<br>2024 年 5 月 21 日<br>2024 Y 5 M 21 D
实验动物设施意见<br>Opinion from laboratory animal facility<br><br>同意<br><br>设施负责人签（章）：<br>Signature （stamp）of the facility Director<br>2024 年 5 月 21 日<br>2024 Y 5 M 21 D

福利伦理委员会审批意见
Approval pinion of Committee

审查委员会表决：
Inspection by menbers：Agree（ √ ）；Disagree（ ）

主任委员签（章）：
Signature （stamp） of Chirman of Committee
2024 年 5 月 27 日
2024 Y 5 M 27 D

备注： □初审； □第 次审查。
Remarks □first trial；□reexamine No.

申报说明：申报时，请提交本表一式两份及电子版。受理文号和批准文号由伦理委员会填写。
Notice:Submitting the Application Format in duplicate and a electronic edition.
The Appl.No. are made out by Jury.
