# Supplementary material for: Exploring the role of RCEO in macrophage-mediated modulation of pulmonary fibrosis
Source: Chin Med. 2026 May 29;21:153. doi: 10.1186/s13020-026-01398-w (PMC13220479; doi:10.1186/s13020-026-01398-w)
Supplement: Supplementary file 2 — Additional file 2. [file 13020_2026_1398_MOESM2_ESM.docx]

1.Western blot

p-PI3K (85 kDa) + PI3K (85 kDa)


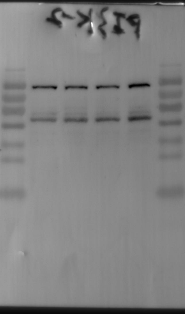

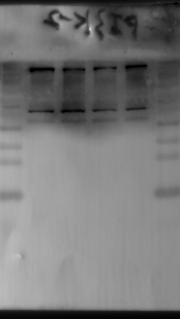


p-Akt (56 kDa) + Akt (56 kDa)


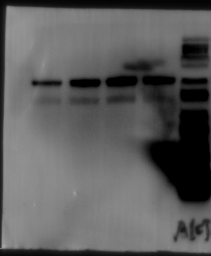

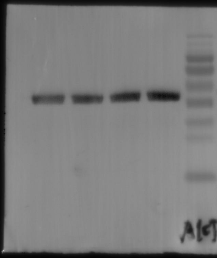


β-Catenin (85 kDa) + β-actin (42 kDa)


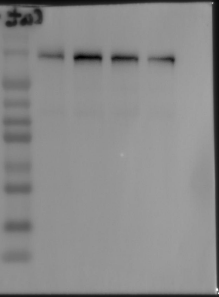

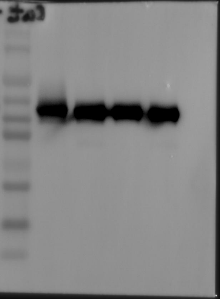


TLR4 (96 kDa) + β-actin (42 kDa)


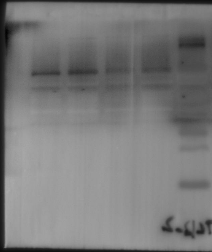

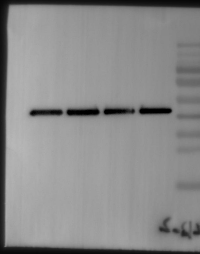


MyD88 (33 kDa) + β-actin (42 kDa)


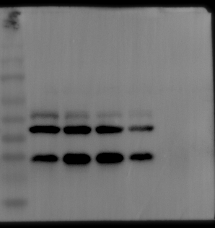

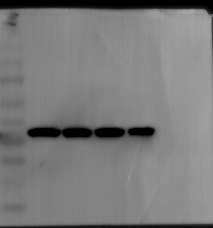


p-NF-κB (65 kDa) + NF-κB (65 kDa)


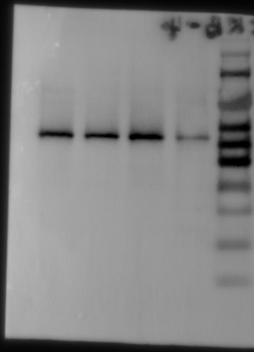

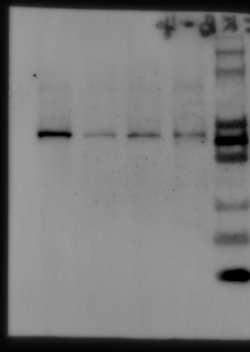


ARG1 (35 kDa) + β-actin (42 kDa)


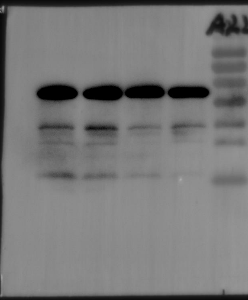

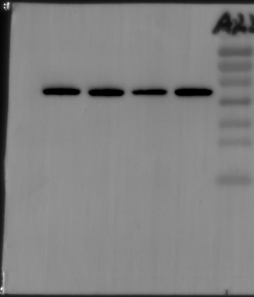


iNOS (65 kDa) + β-actin (42 kDa)


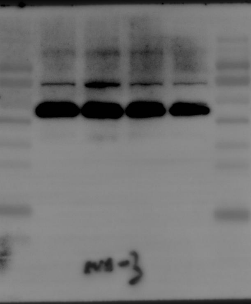

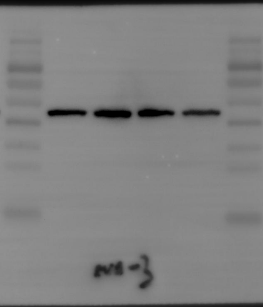


IL-10 (21 kDa) + β-actin (42 kDa)


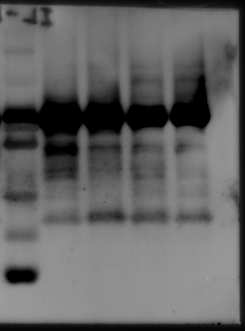

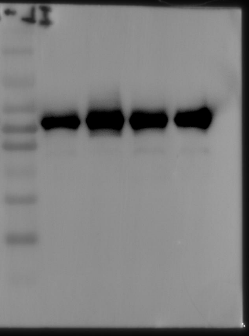


TNF-α (26 kDa) + β-actin (42 kDa)


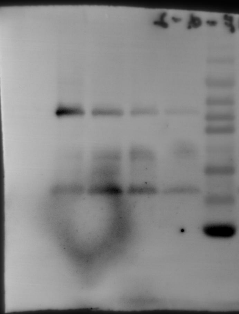

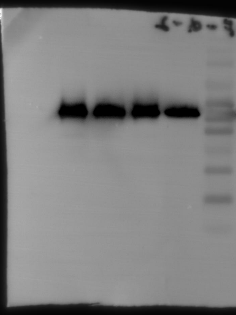


E-cadherin (140 kDa) + β-actin (42 kDa)


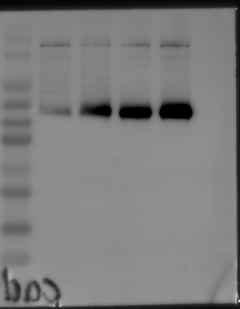

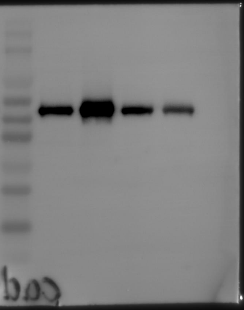


α-SMA (42 kDa) + β-actin (42 kDa)


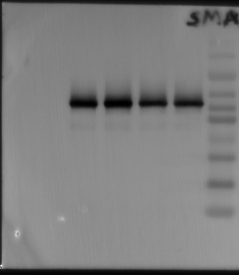

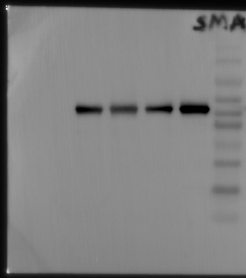


Collagen-Ⅰ (139 kDa) + β-actin (42 kDa)


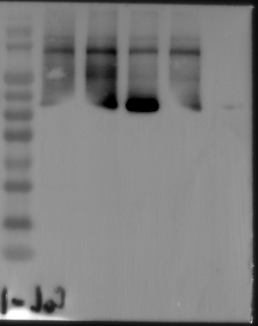

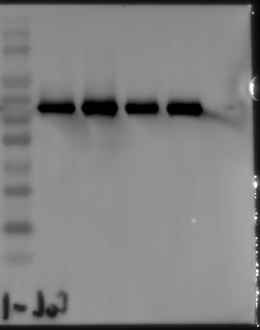


Fibronectin (272 kDa) + β-actin (42 kDa)


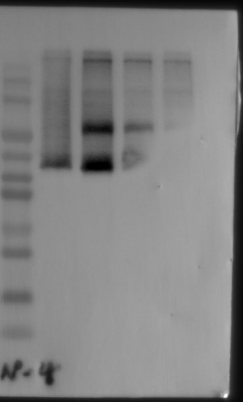

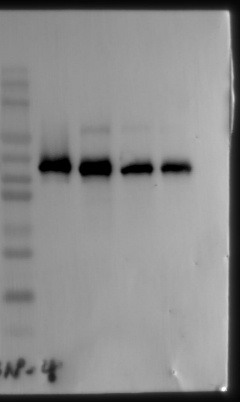


2.Molecular Docking

PI3K-Camphor (binding energy=-5.3, with hydrogen bonds)


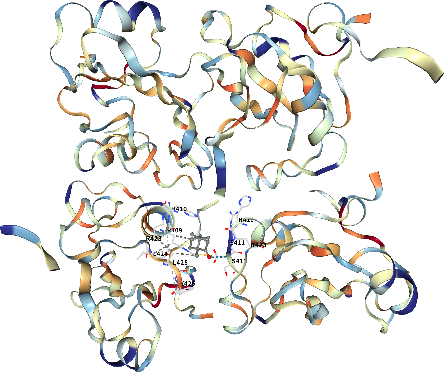

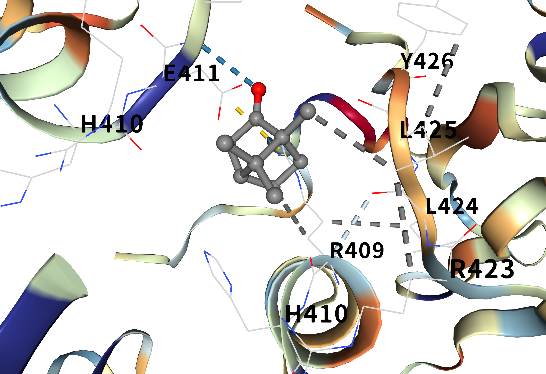


PI3K-Cyclohexene (binding energy=-6.8, without hydrogen bonds)


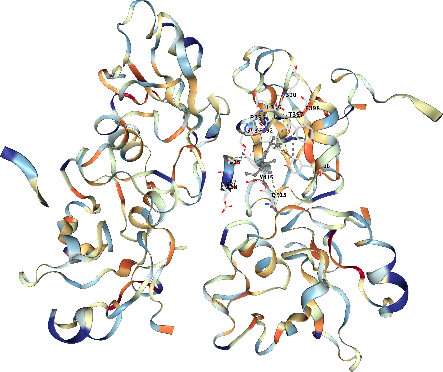

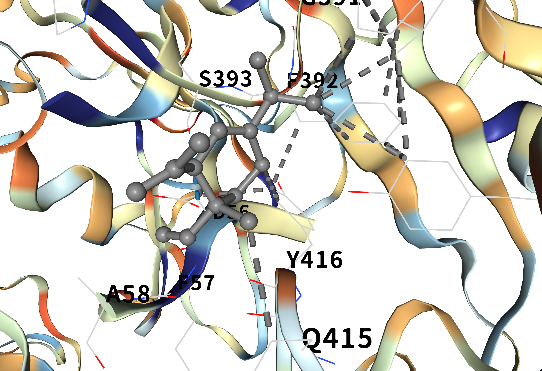


PI3K-BETA-ELEMENE (binding energy=-5.7, without hydrogen bonds)


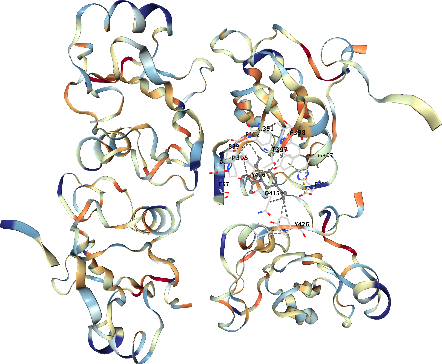

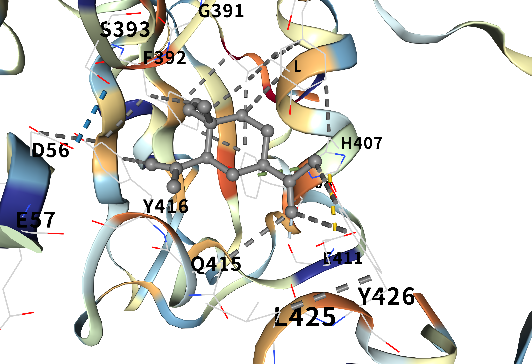


PI3K- BETA-CARYOPHYLLENE (binding energy=-6.6, without hydrogen bonds)


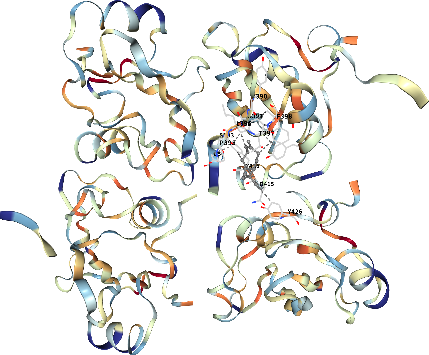

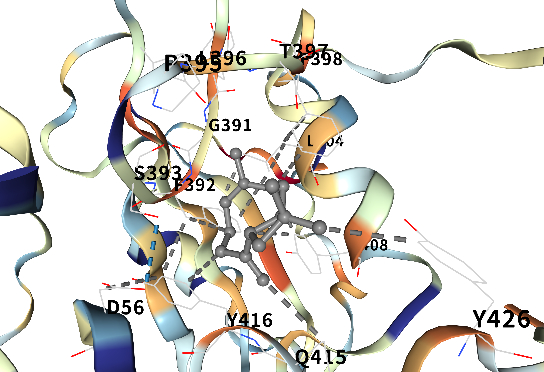


PI3K-gamma-Muurolene (binding energy=-6.1, without hydrogen bonds)


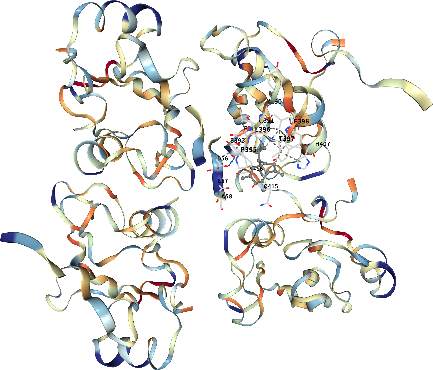

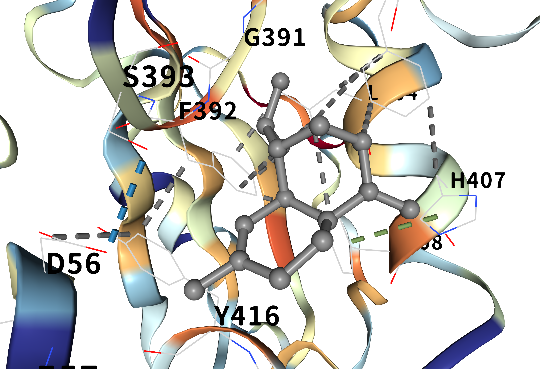


PI3K-gamma-Elemene (binding energy=-5.9, without hydrogen bonds)


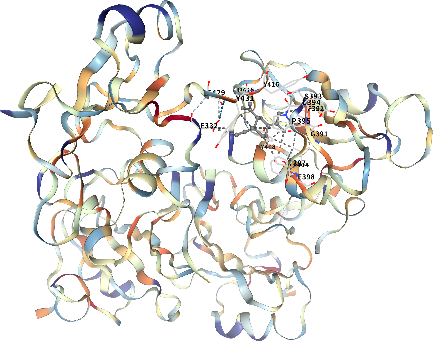

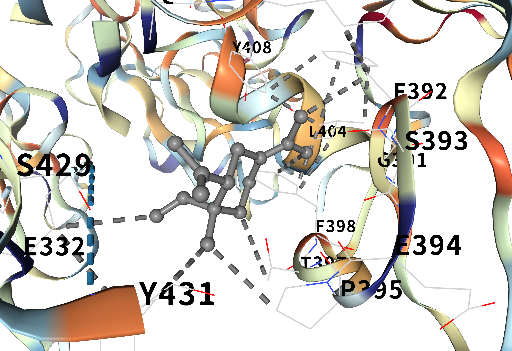


PI3K-BETA-SELINENE (binding energy=-6.5, without hydrogen bonds)


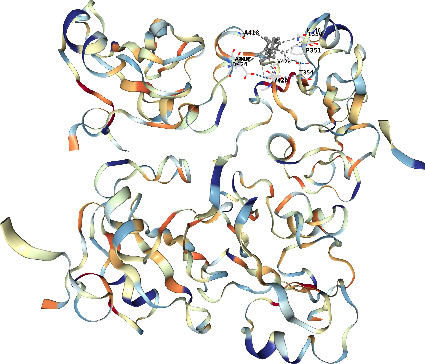

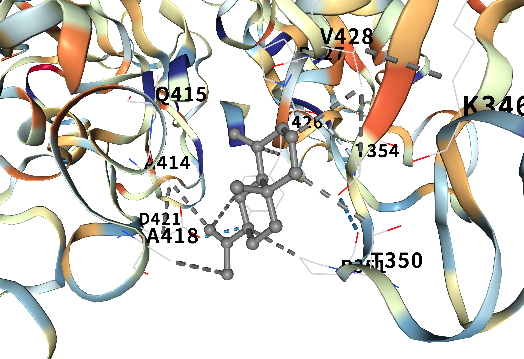


PI3K-Curzerene (binding energy=-6.4, without hydrogen bonds)


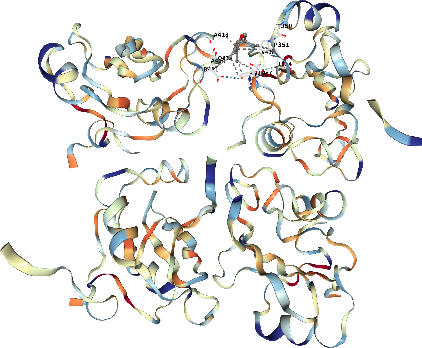

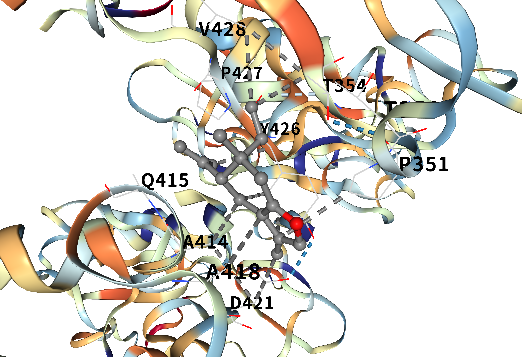


PI3K-Germacrone (binding energy=-6.9, with hydrogen bonds)


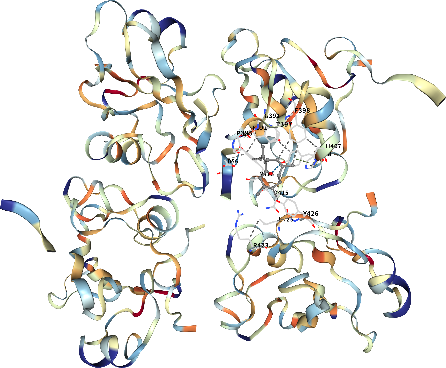

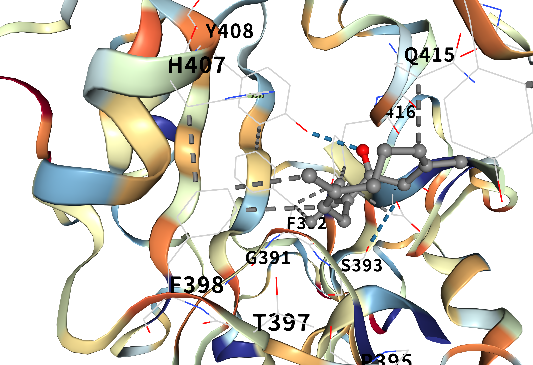


PI3K-beta-Elemenone (binding energy=-6.5, with hydrogen bonds)


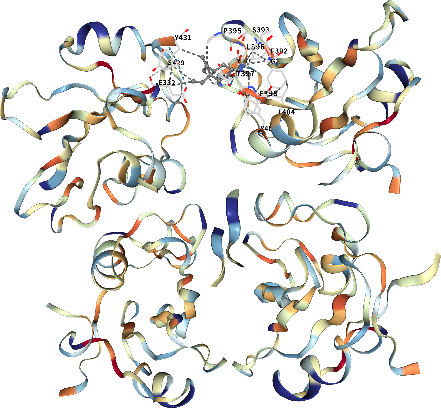

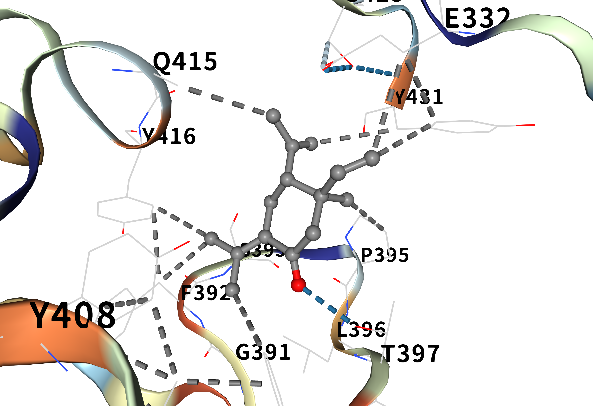


PI3K-Chiapin B (binding energy=-8.3, with hydrogen bonds)


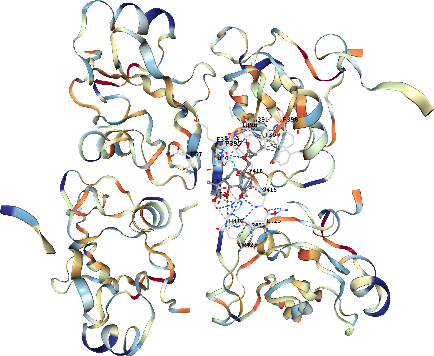

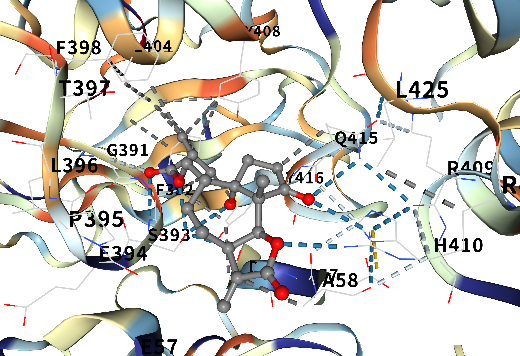


PI3K-Hydroxyvalerenic Acid (binding energy=-7.2, with hydrogen bonds)


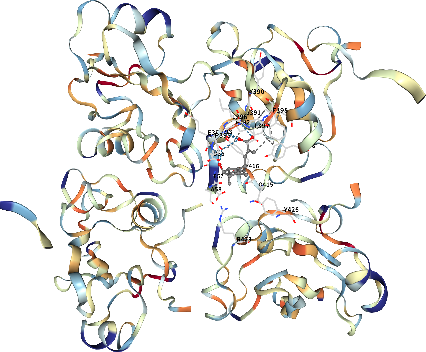

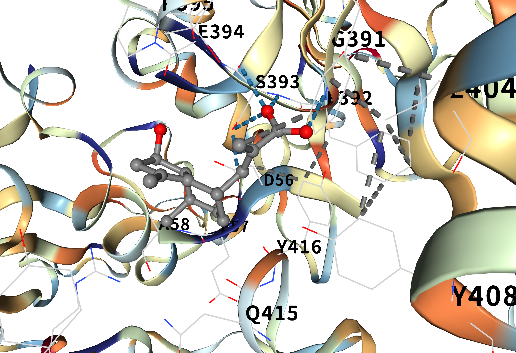


PI3K-beta-Cyclocostunolide (binding energy=-7.1, with hydrogen bonds)


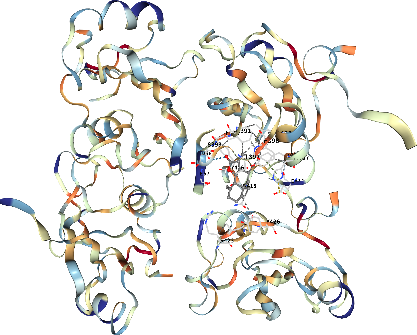

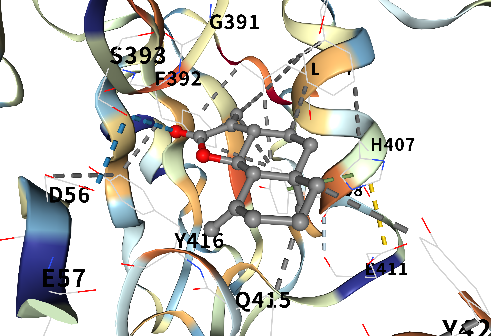


PI3K-Velleral (binding energy=-6.5, with hydrogen bonds)


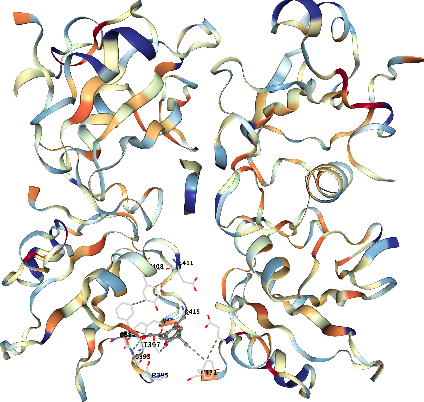

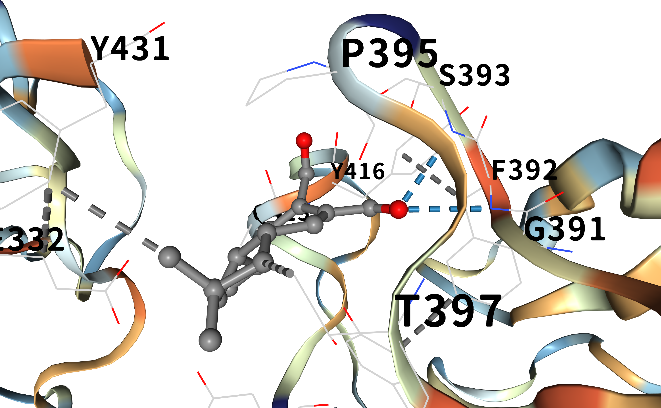


PI3K-Cyclobutane, tetrakis(1-methylethylidene)- (binding energy=-6.2, without hydrogen bonds)


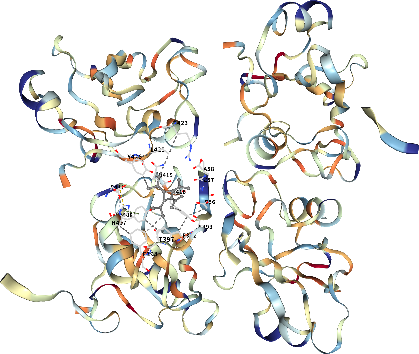

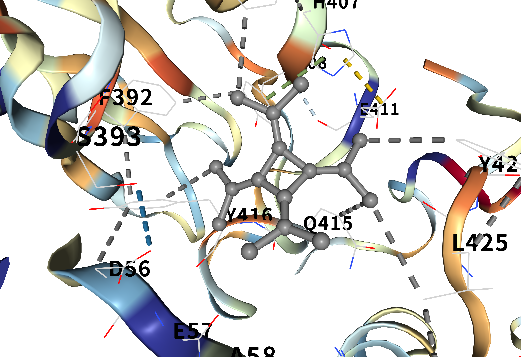


PI3K-(+/-)-5c,8c-dihydroxy-4a-methyl-(4ar,4bt,8at)-4,4a,4b,5,6,7,8,8a,9,10-decahydro-3H-phenanthren-2-one (binding energy=-8, with hydrogen bonds)


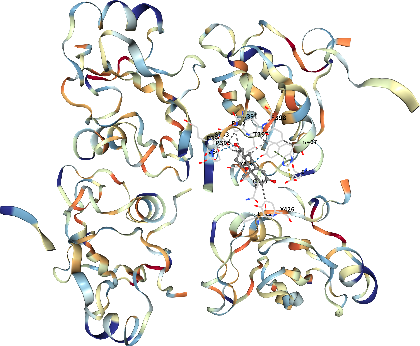

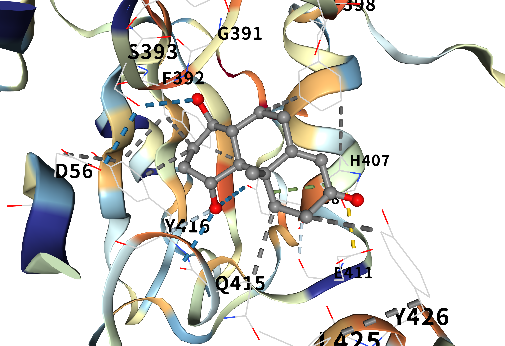


PI3K-Alantic anhydride (binding energy=-6, with hydrogen bonds)


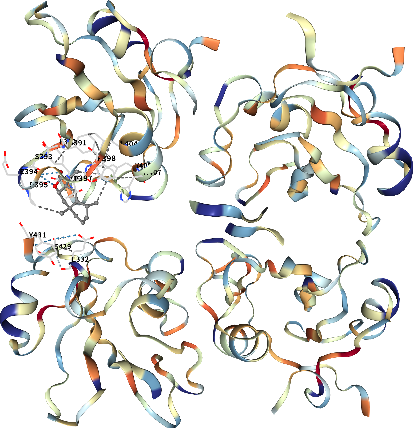

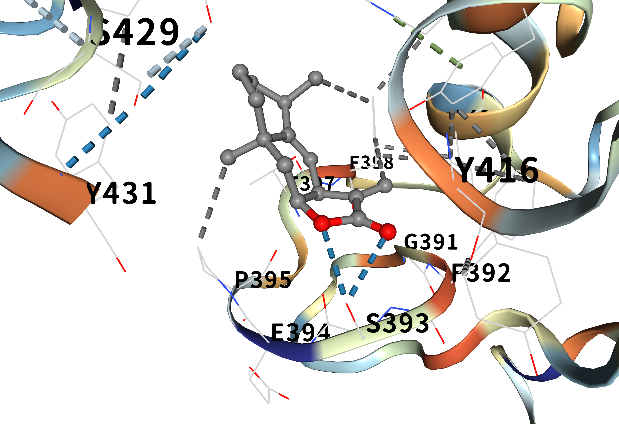


TLR4-Camphor (binding energy=-5.3, without hydrogen bonds)


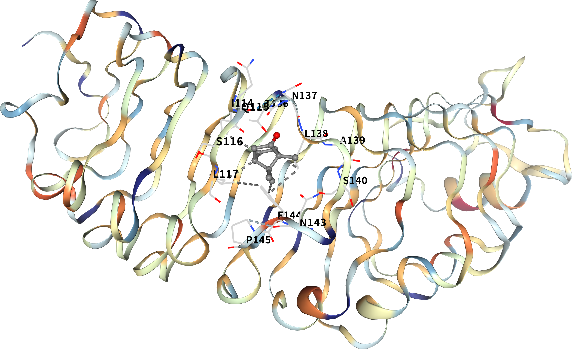

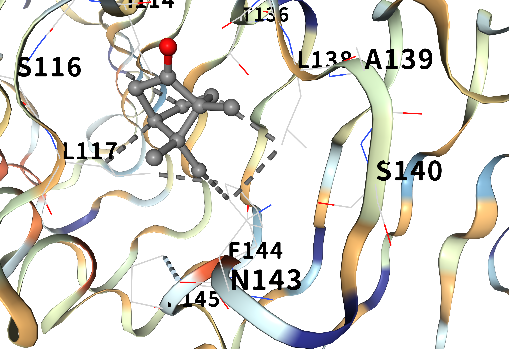


TLR4-Cyclohexene (binding energy=-5.6, without hydrogen bonds)


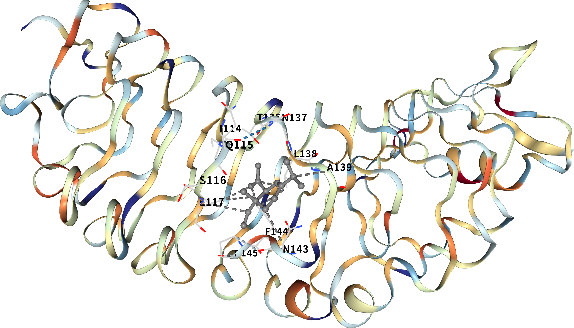

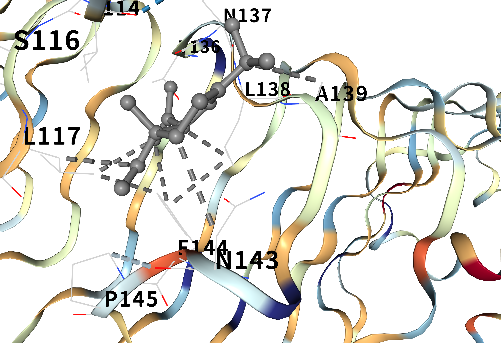


TLR4-BETA-ELEMENE (binding energy=-6.1, without hydrogen bonds)


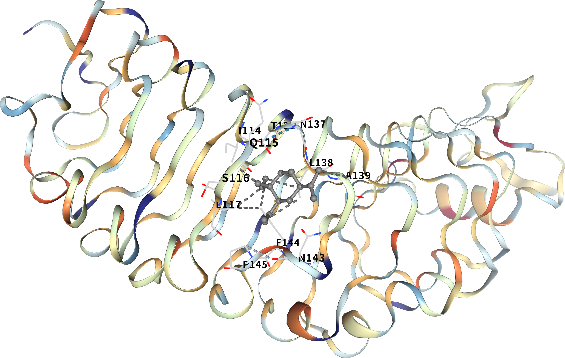

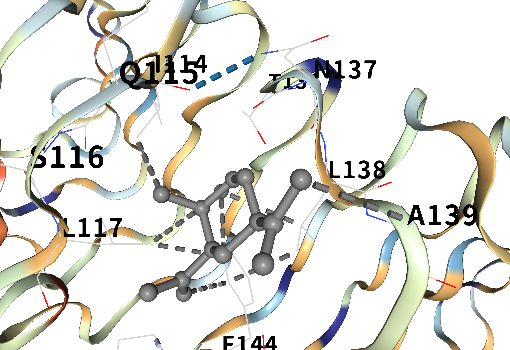


TLR4-BETA-CARYOPHYLLENE (binding energy=-6, without hydrogen bonds)


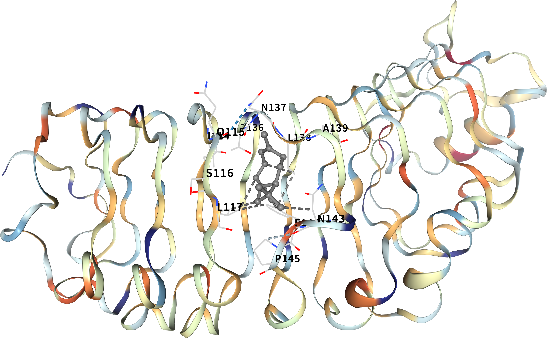

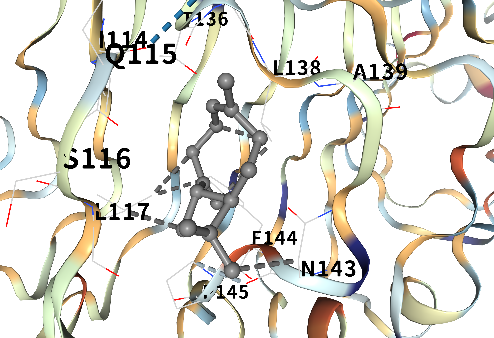


TLR4-gamma-Muurolene (binding energy=-6.8, without hydrogen bonds)


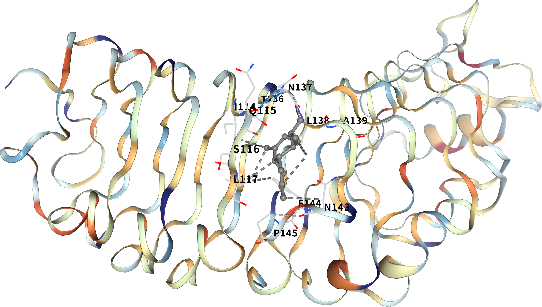

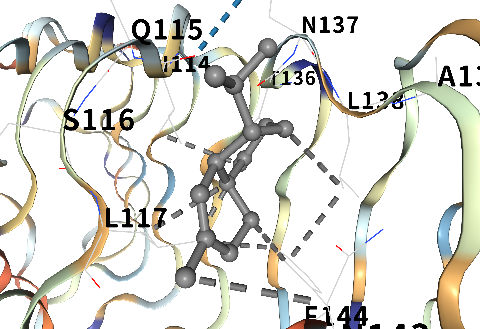


TLR4-gamma-Elemene (binding energy=-5.1, without hydrogen bonds)


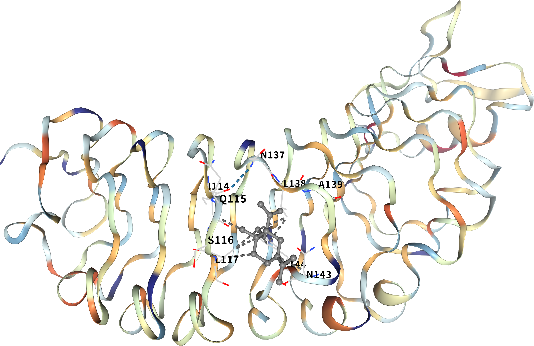

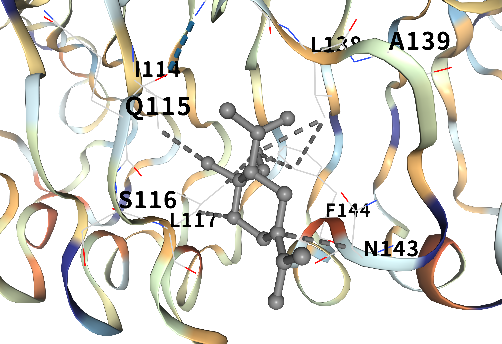


TLR4-BETA-SELINENE (binding energy=-6.2, without hydrogen bonds)


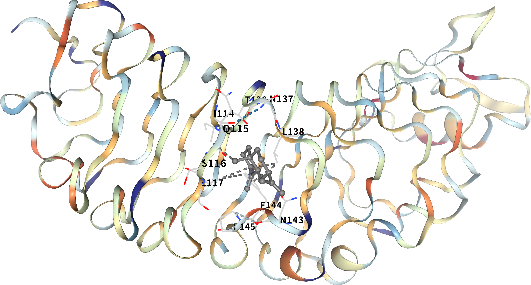

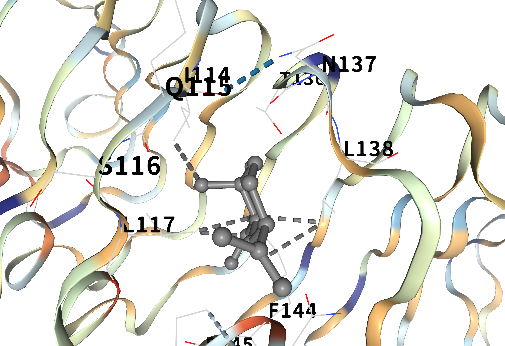


TLR4-Curzerene (binding energy=-5.4, without hydrogen bonds)


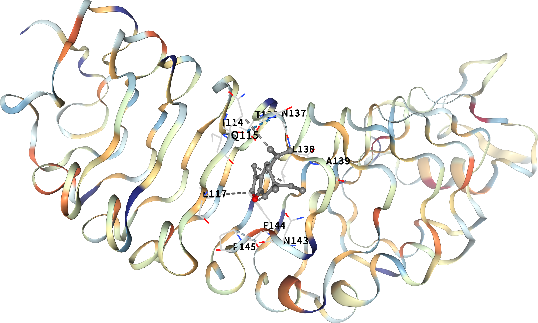

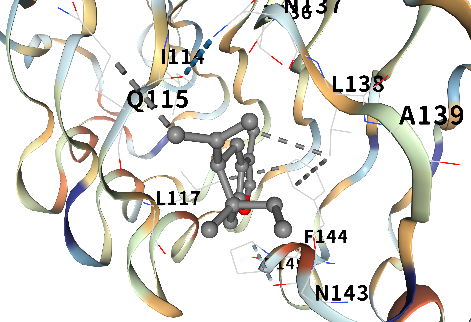


TLR4-Germacrone (binding energy=-6.4, with hydrogen bonds)


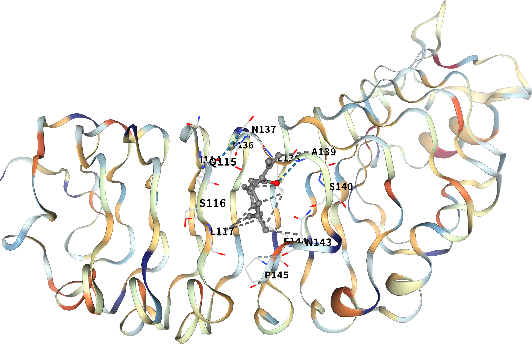

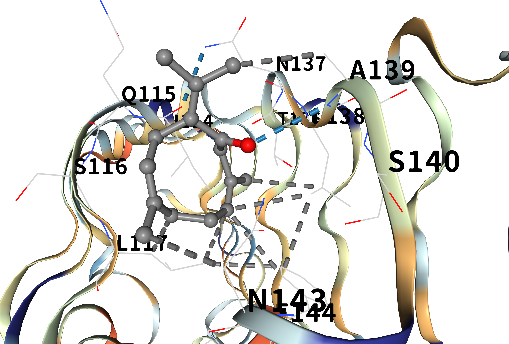


TLR4-beta-Elemenone (binding energy=-5.5, without hydrogen bonds)


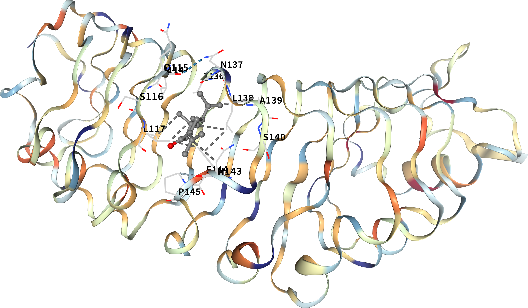

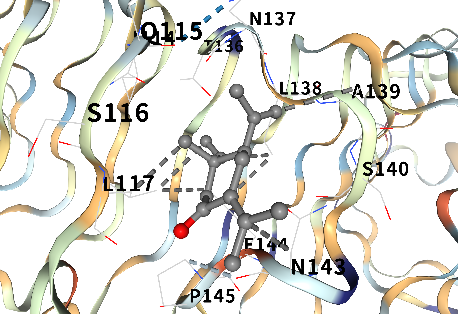


TLR4-Chiapin B (binding energy=-6.2, with hydrogen bonds)


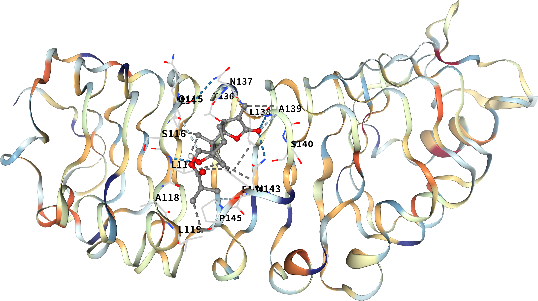

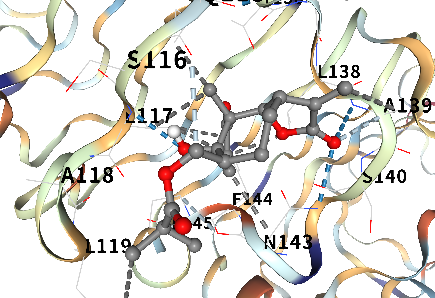


TLR4-Hydroxyvalerenic Acid (binding energy=-6.3, with hydrogen bonds)


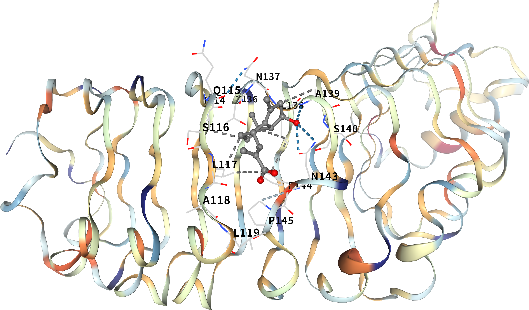

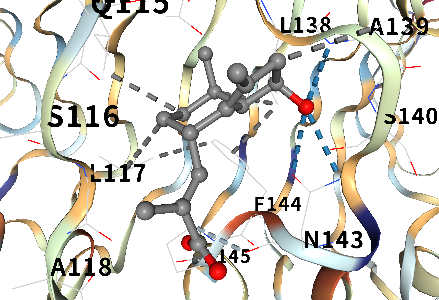


TLR4-beta-Cyclocostunolide (binding energy=-6.5, without hydrogen bonds)


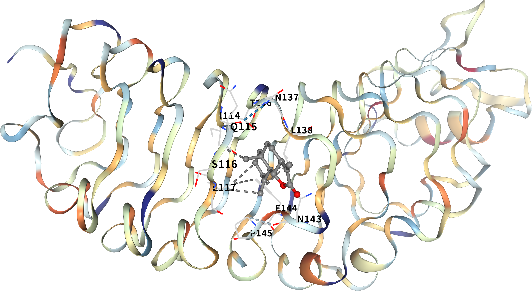

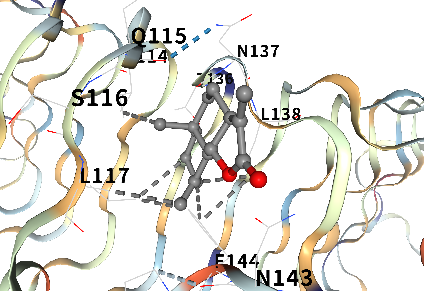


TLR4-Velleral (binding energy=-6, with hydrogen bonds)


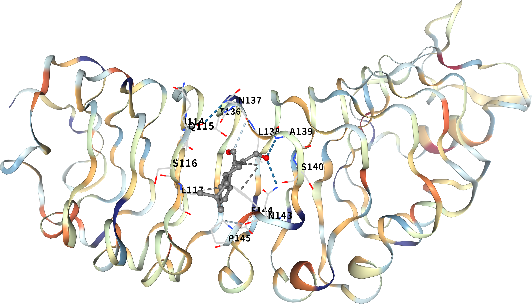

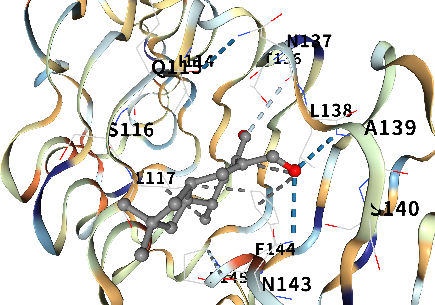


TLR4-Cyclobutane, tetrakis(1-methylethylidene)- (binding energy=-5.5, without hydrogen bonds)


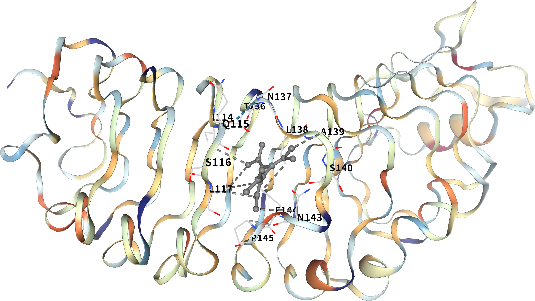

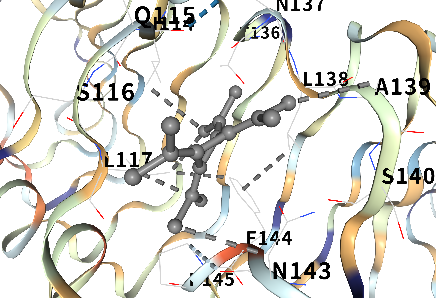


TLR4-(+/-)-5c,8c-dihydroxy-4a-methyl-(4ar,4bt,8at)-4,4a,4b,5,6,7,8,8a,9,10-decahydro-3H-phenanthren-2-one (binding energy=-6.2, with hydrogen bonds)


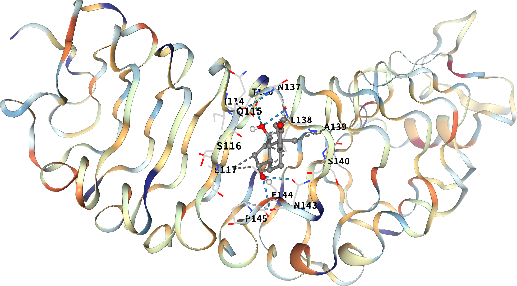

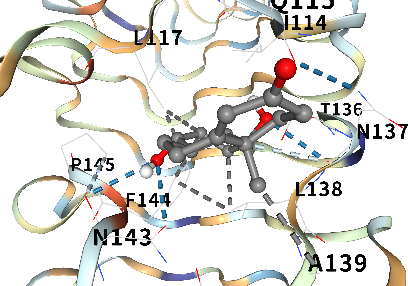


TLR4- Alantic anhydride (binding energy=-6.2, without hydrogen bonds)


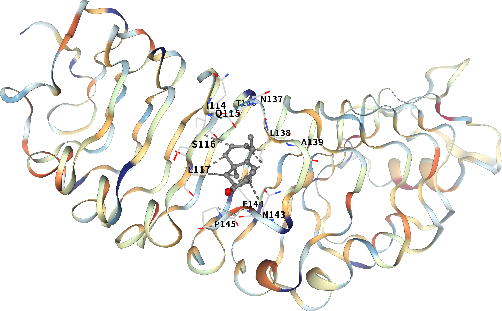

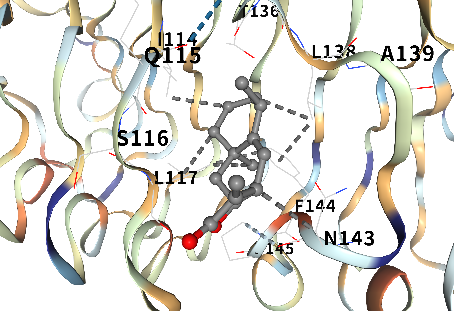


3. Chemical structure of the main compounds in lung

| 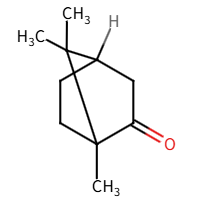 | 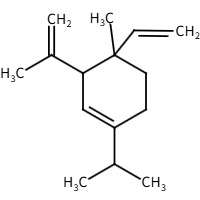 | 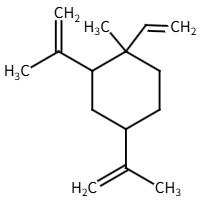 | 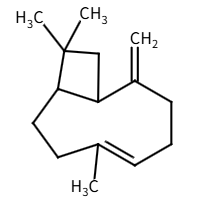 |
| --- | --- | --- | --- |
| 1 Camphor | 2 Cyclohexene | 3 BETA-ELEMENE | 4 BETA-CARYOPHYLLENE |
|  |  |  |  |
| 5 gamma-Muurolene | 6 gamma-Elemene | 7 BETA-SELINENE | 8 Curzerene |
|  |  |  |  |
| 9 Germacrone | 10 beta-Elemenone | 11 Chiapin B | 12 Hydroxyvalerenic Acid |
|  |  |  |  |
| 13 beta-Cyclocostunolide | 14 Velleral | 15 Cyclobutane, tetrakis(1-methylethylidene)- | 16 (+/-)-5c,8c-dihydroxy-4a-methyl-(4ar,4bt,8at)-4,4a,4b,5,6,7,8,8a,9,10-decahydro-3H-phenanthren-2-one |
|  |  |  |  |
| 17 Alantic anhydride |  |  |  |

4. Screening of Drug Concentrations in Cell Experiments

RCEO was dissolved in complete medium (containing 10% fetal bovine serum, FBS) to prepare drug-containing medium at the following concentrations: 2.5, 5, 10, 20, 40, 80, 160, and 320 μg/mL. The prepared medium was co-cultured with different cell lines (RAW 264.7 cells, MLE-12 cells, and 3T3-L1 cells) for 6, 12, and 24 hours. Cell viability was assessed using the CCK-8 assay kit according to the manufacturer’s instructions, and the optical density (OD) values were measured. The results demonstrated that RCEO exhibited negligible cytotoxicity toward all tested cell lines at 6 and 12 hours. However, after 24 hours of treatment, RCEO-160 and RCEO-320 exerted significant cytotoxic effects on RAW 264.7 cells and MLE-12 cells, which could potentially interfere with experimental outcomes; therefore, these concentrations were excluded. In contrast, RCEO-20 already exhibited toxicity toward 3T3-L1 cells, significantly inhibiting fibroblast growth. Consequently, the following concentrations were selected for subsequent cell experiments: RCEO-20, RCEO-40, and RCEO-80 (Fig. S1).

Fig. S1. Effects of RCEO on the cell proliferation of RAW 264.7, MLE-12, and 3T3-L1 Cells at Different Concentrations and Incubation Times. (A–C) Effects of varying RCEO concentrations on the cell proliferation of RAW 264.7 cells after 6, 12, and 24 hours of incubation (n=6). (D–F) Effects of varying RCEO concentrations on the cell proliferation of MLE-12 cells after 6, 12, and 24 hours of incubation (n=6). (G–I) Effects of varying RCEO concentrations on the cell proliferation of 3T3-L1 cells after 6, 12, and 24 hours of incubation (n=6). Data analysis was performed using one-way ANOVA and data were presented as mean ± SD. **P* < 0.05, ***P* < 0.01.

5. Effect of RCEO on Ccl2 Concentration in the Supernatant of RAW 264.7 Cells

To investigate the effect of RCEO on the concentration of Ccl2 in the supernatant of RAW 264.7 cells, the cells were co-incubated with RCEO and RS 09 for 24 hours. The experimental groups were as follows: Control group, BLM group, BLM+RCEO group, and BLM+RCEO+RS 09 group. After incubation, the cell suspension was collected using a pipette. The supernatant was obtained by centrifugation at 3500 rpm for 10 minutes at 4°C. The concentration of Ccl2 in the cell supernatant of each group was measured according to the manufacturer's instructions of the mouse Ccl2 ELISA kit (mlbio, China). The results are shown in Fig. S2. The concentration of Ccl2 in the supernatant of the control group was low, remaining at a normal level. In contrast, co-incubation with BLM led to an approximately 6-fold increase in Ccl2 concentration. The Ccl2 concentration in the supernatant of the BR group was rapidly reduced, whereas it was significantly increased in the BRR group. These results indicate that RCEO inhibits the release of Ccl2 from RAW 264.7 cells, and this inhibitory effect can be reversed by the addition of RS 09.

Figure S2. Effect of RCEO on Ccl2 concentration in the supernatant of RAW 264.7 cells. Data analysis was performed using one-way ANOVA and data were presented as mean ± SD. **P* < 0.05, ***P* < 0.01.
